# Cellular Senescence Is a Central Driver of Cognitive Disparities in Aging

**DOI:** 10.1111/acel.70041

**Published:** 2025-03-12

**Authors:** Matthew P. Baier, Rojina Ranjit, Daniel B. Owen, Jenna L. Wilson, Megan A. Stiles, Anthony M. Masingale, Zachary Thomas, Anne Bredegaard, David M. Sherry, Sreemathi Logan

**Affiliations:** ^1^ Department of Biochemistry and Physiology University of Oklahoma Health Sciences Oklahoma City Oklahoma USA; ^2^ Center for Geroscience and Healthy Brain Aging University of Oklahoma Health Sciences Oklahoma City Oklahoma USA; ^3^ Department of Cell Biology University of Oklahoma Health Sciences Oklahoma City Oklahoma USA; ^4^ Neuroscience Program University of Oklahoma Health Sciences Oklahoma City Oklahoma USA

**Keywords:** cellular senescence, cognitive heterogeneity, dementia, neuroinflammation, reactive gliosis, senolytic

## Abstract

Cognitive function in aging is heterogeneous: while some older individuals develop significant impairments and dementia, others remain resilient and retain cognitive function throughout their lifespan. The molecular mechanisms that underlie these divergent cognitive trajectories, however, remain largely unresolved. Here, we utilized a high‐resolution home‐cage‐based cognitive testing paradigm to delineate mechanisms that contribute to age‐related cognitive heterogeneity. We cognitively stratified aged C57Bl/6N male mice by cognitive performance into intact (resilient) or impaired subgroups based on young performance benchmarks. Cognitively impaired males exhibited marked reactive gliosis in the hippocampus, characterized by microglial activation, increased astrocyte arborization, and elevated transcriptional expression of reactivity markers. These changes were accompanied by increased markers of cellular senescence and the associated senescence‐associated secretory phenotype (SASP) in impaired animals, including p16^INK4a^, SASP factors (e.g., *Il‐6*, *Il‐1b*, *Mmp3*), and SA‐β‐gal staining in the hippocampus. Notably, clearance of senescent cells using senolytic agents dasatinib and quercetin ameliorated the heterogeneity in cognitive performance observed with age and attenuated impairment‐associated gliosis, senescence markers, and mitochondrial dysfunction. Aged female mice could not be stratified into subgroups yet showed increased neuroinflammation with age that was not resolved with senolytics. Collectively, our findings implicate cellular senescence as a central driver of sex‐specific neuroinflammation that drives divergent cognitive trajectories in aging. Thus, we demonstrate that senolytic treatment is an effective therapeutic strategy to mitigate cognitive impairment by reducing neuroinflammation and associated metabolic disturbances.

## Graphical abstract

The molecular underpinnings of divergent cognitive trajectories in aging remain unresolved. Using high‐resolution behavioral testing to cognitively stratify aged male mice into ‘intact’ and ‘impaired’ subgroups, we show that reactive gliosis and cellular senescence are key determinants of disparities in cognitive function with age.

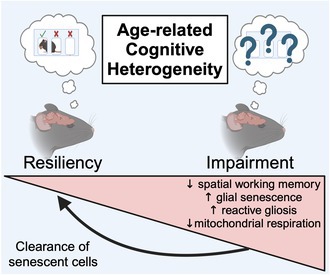

## 1 Introduction

As individuals age, progressive deterioration of normal physiology increases vulnerability to multiple pathologies, including age‐related cognitive decline, dementia, and neurodegenerative diseases (López‐Otín et al. 2013). However, cognitive decline in aging is notably heterogeneous (Marron et al. 2019). While some individuals exhibit profound cognitive deficits, others exhibit resilience and maintain robust function throughout their lifespan. Despite the recognition of large disparities apparent in cognitive function (e.g., cognitive heterogeneity) among individuals with age, most rodent models of aging do not adequately capture the full spectrum of cognitive outcomes, largely due to the lack of sensitivity of current preclinical cognitive testing paradigms. Consequently, mechanistic studies aimed at elucidating the molecular drivers of age‐related cognitive heterogeneity have been limited.

Reactive gliosis and neuroinflammation are well‐documented outcomes associated with cognitive impairment in both aging and Alzheimer's disease (AD) (Heneka et al. 2024; Salas et al. 2020). Glial cells (e.g., astrocytes and microglia) critically influence cognitive health by altering their repertoire of secreted factors (Heneka et al. 2024). Upon activation, these cells undergo morphological and structural alterations constituting reactive gliosis, releasing cytokines that can confer protective effects under acute or injury‐related conditions (Sochocka et al. 2017). Sustained reactive gliosis, however, fosters elevated levels of proinflammatory cytokines and chemokines, ultimately thought to drive age‐ and neurodegenerative disease‐associated cognitive dysfunction. While glial activation is frequently observed in aging and neurodegenerative diseases, the drivers of the sustained glial activation in aging and their specific role in determining cognitive trajectories with age remain unclear.

One mechanism thought to induce reactive gliosis in aging and neurodegenerative diseases is the accumulation of senescent cells and their resulting proinflammatory secretome. Cellular senescence, an irreversible growth arrest induced by cellular damage [e.g., telomere shortening, DNA and oxidative damage (Chaib et al. 2022)], is characterized by evasion of apoptosis and immune cell‐mediated clearance. Senescent cells undergo a conglomerate of tissue‐specific alterations, including upregulation of cyclin‐dependent kinase inhibitors (e.g., p16^INK4a^, p21^WAF1^) and secretion of proinflammatory products collectively termed the senescence‐associated secretory phenotype (SASP) (Huang et al. 2022). SASP factors include cytokines (e.g., IL‐6, TNFα), chemokines, extracellular matrix proteases, remodeling factors, and bioactive lipids, among others (Fitsiou et al. 2021; Tchkonia and Kirkland 2018; Wiley and Campisi 2021). While cell senescence can play protective roles (e.g., tumor suppression), the accumulation of senescent cells with age and persistent SASP expression significantly contributes to age‐associated tissue dysfunction (Yousefzadeh et al. 2020).

Senolytic therapeutics (e.g., dasatinib and quercetin, fisetin, navitoclax [ABT‐263], etc.) target the anti‐apoptotic pathways upregulated by senescent cells, thereby facilitating targeted clearance of senescent cells and attenuation of SASP‐induced inflammation (Zhang et al. 2022). Notably, senolytic treatment in AD models has shown improvements in disease pathophysiology, including reactive gliosis and amyloid beta (Aβ)/tau‐induced glial cell senescence (Bussian et al. 2018; Zhang et al. 2019). However, high expression of pathogenic mediators of AD (Aβ and tau) beginning early in life that induce senescence phenotypes in multiple brain cell types precludes the identification of the effects of age alone. Given that advanced age is the strongest risk factor for AD onset and progression, dissecting the contribution of normal aging to the induction of senescent cells in the brain remains a critical area for further investigation. Although some studies have reported modest senolytic‐mediated benefits on spatial memory in non‐AD models of aging (Ogrodnik et al. 2021), classical behavior assays may be insufficiently sensitive to discriminate mechanisms of healthy brain aging from cognitive impairment. Consequently, the specific contribution of cellular senescence to age‐related cognitive heterogeneity is still not well defined.

To address these issues, our laboratory has established a rigorous, high‐resolution cognitive testing approach using an automated home‐cage‐based data analysis system (PhenoTyper) (Baier et al. 2022; Grieco et al. 2021; Logan et al. 2023, 2018, 2019). By benchmarking performance in young adult male mice (5–7 months), we derived a statistical cutoff of 1000 entries to reach an 80% success rate during the reversal learning task of this paradigm to reliably classify aged mice based on performance as either cognitively “intact” or “impaired” (Logan et al. 2023). This stratification methodology provides an innovative and statistically powerful approach to investigate the molecular and cellular mechanisms of aging specific to cognitive impairment. We have previously demonstrated that aged C57Bl/6N male mice exhibit variations in cognitive performance comparable to those observed in aging humans, with one subset showing preserved function and another displaying significant deficits in spatial working memory (Logan et al. 2023). Here, we report that the increased burden of reactive gliosis and cellular senescence in the hippocampus delineates the cognitive status of aged C57Bl/6N male mice. Moreover, treatment with the senolytic regimen, dasatinib and quercetin (D + Q), ameliorates the observed heterogeneity in spatial working memory and attenuates cellular senescence markers and associated pathological signatures, including reactive gliosis and mitochondrial function deficits. These findings provide the first evidence that heterogeneity in gliosis and senescence burden is a key determinant of disparities in cognitive trajectories in normative brain aging.

## 2 Results

### 2.1 High‐Resolution Performance‐Based Cognitive Stratification of Aged Mice

Disparities in cognitive function in aging (e.g., cognitive heterogeneity) are well documented in humans and higher‐order mammals. However, traditional preclinical cognitive testing paradigms (e.g., radial arm water maze) are often less effective at differentiating variations in cognitive performance within aged animals (Figure S1) due to the limited number of trials per day and the influence of diurnal activity patterns on behavior, among other factors. To overcome these challenges and investigate the molecular underpinnings of age‐related cognitive heterogeneity in a rodent model, we utilized the high‐resolution home‐cage testing paradigm (PhenoTyper) previously validated by our lab (Baier et al. 2022; Logan et al. 2023) to assess spatial working memory in aged male and female C57Bl/6N mice (22‐24mo).

**FIGURE 1:**
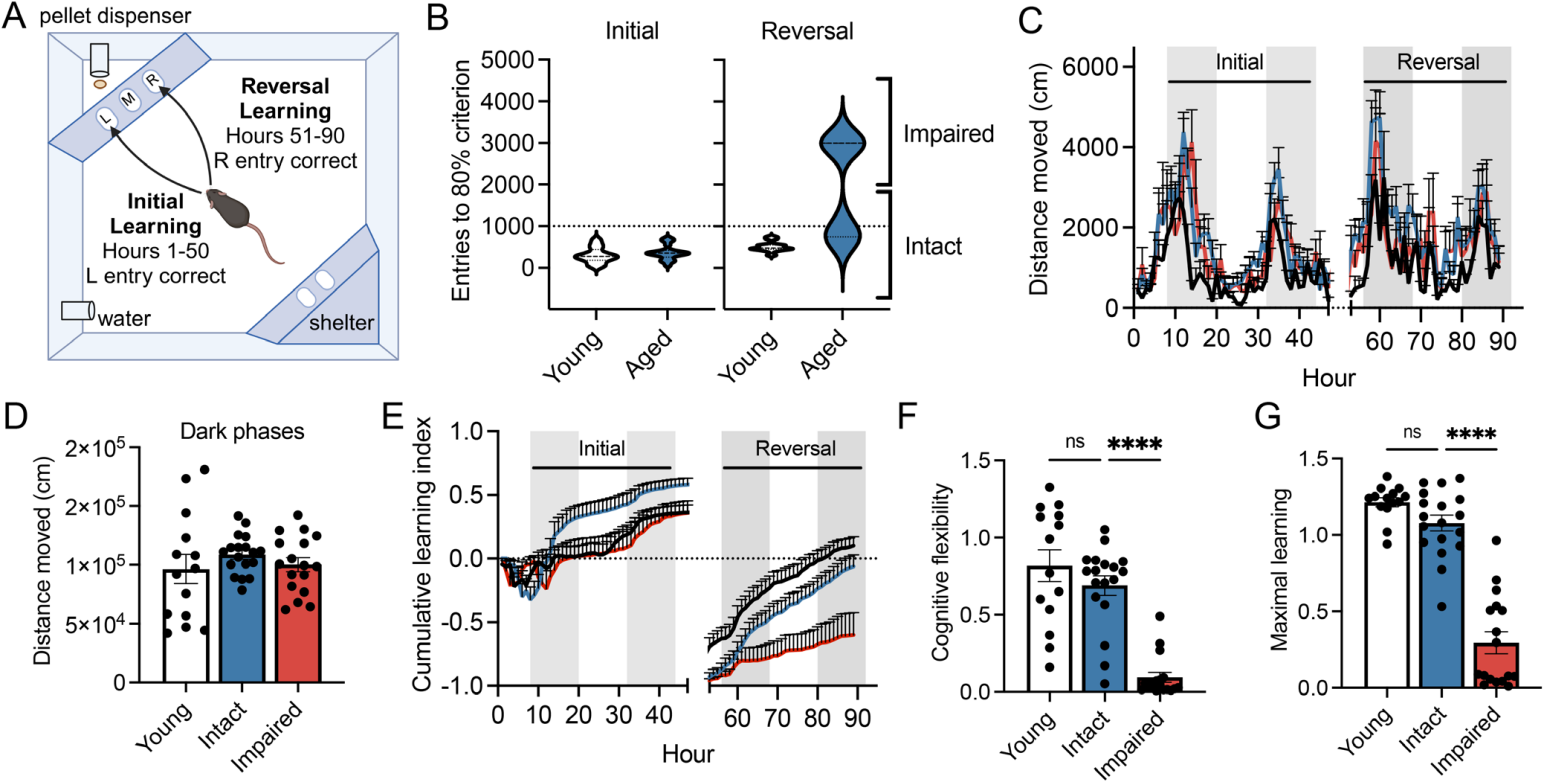
High‐resolution performance‐based spatial working memory paradigm facilitates cognitive stratification of aged male mice. (A) Illustration of the PhenoTyper with CognitionWall used to assess cognitive performance over a 90‐h period. Initial discrimination learning occurs over the first 2 days of testing (hours 1–50), where the mouse learns to enter the left‐most entry of the cognition wall five times to receive a food pellet. On days 3–4 (hours 51–90), the mouse undergoes a reversal learning task, relearning to enter the right‐most entry to receive a food pellet. Behavior is assessed during both dark and light periods using an infrared camera, with movements tracked using Noldus Ethovision software. (B) Entries needed to reach the 80% criterion (based off the success rate of the trailing 30 entries) in the initial learning and reversal learning tasks of young (6 months; *n* = 14) and aged (22–24 months; *n* = 35) mice. (C) Circadian activity assessed via the onset of activity during the dark phase was comparable among young and cognitively stratified aged groups. (D) Total distance moved during the dark phases of the day/light cycle was unchanged between groups. (E) Cumulative learning index depicts separations between aged cognitively intact and impaired animals during the reversal phase. (F) Cognitive flexibility was assessed during the first 10 h of the reversal phase and was significantly reduced in the aged cognitively impaired group. (G) Maximal learning of aged cognitively impaired mice was significantly decreased at the end of the reversal phase. For graphs C–N, colors represent the following: Young (black), aged intact (blue), aged impaired (red). Shaded bars in graphs C and E represent the dark periods of the L:D cycle. Error bars: Mean ± SEM. *p < 0.05, **p < 0.01, ***p < 0.001, ****p < 0.0001.

Using this paradigm, spontaneous behaviors of mice were recorded continuously over 90 h, encompassing both light and dark phases of the light/dark cycle. Mice initially learn to enter one of three holes for a food reward (initial discrimination, hours 1–50) on a fixed ratio 5 schedule, followed by a reversal learning task (hours 51–90) that requires them to relearn to enter a different hole (Figure 1A). Spatial learning is assessed for each hour and visualized as a cumulative learning index (calculated as the ratio of correct to incorrect entries) that is compounded hourly for both initial discrimination and reversal phases. Cognitive performance is evaluated using three key metrics: the number of entries required to reach an 80% success criterion, cognitive flexibility, and maximal learning.

The number of entries required to achieve an 80% success rate in the trailing 30 entries was calculated separately for the initial learning and reversal phases. This 80% criterion has been validated in discriminating cognitive performance in aged male C57Bl/6 mice (Logan et al. 2018) in addition to models of AD (Remmelink, Smit, et al. 2016) and Duchenne muscular dystrophy (Remmelink, Aartsma‐Rus, et al. 2016). Mice that failed to reach this criterion were assigned a value of 3000 entries, allowing their data to be visualized alongside mice that met the criterion. Cognitive flexibility, a measure of the ability to extinguish prior learning and relearn during the reversal phase, was assessed as the rate of improvement during the first dark cycle of the reversal phase. Maximal learning was defined as the cumulative index achieved at the end of the reversal phase.

Aged male mice performed comparably to young (5–7 months) reference controls during initial learning, requiring a similar number of entries needed to reach the 80% criterion. However, significant variability in performance emerged during the reversal phase, with some aged males failing to meet the criterion altogether (Figure 1B). To better understand factors driving impairment that are distinct from healthy aging, we stratified this aged cohort into cognitively intact and impaired subgroups based on a threshold of 1000 entries to reach the criterion in the reversal phase. This metric has been previously derived and validated by our lab based on the optimal performance of young males in the reversal (Logan et al. 2023). As such, cognitively impaired aged males required significantly more entries to reach the criterion compared to both young controls and cognitively intact males (Figure 1B).

Subsequent subgroup analysis showed no differences in circadian activity patterns between cognitively stratified intact and impaired aged mice, as measured by the onset of activity and total distance moved during the dark phases (Figure 1C,D). Visualization of the cumulative learning index revealed clear distinctions between the performance of aged subgroups (Figure 1E). Cognitively intact aged mice demonstrated quicker learning in the initial learning phase, while cognitively impaired mice exhibited marked deficits in cognitive flexibility (Figure 1F) and maximal learning (Figure 1G) during the reversal phase.

Like males, aged female mice performed comparably to young controls in the initial learning task, as determined by the number of entries to achieve the 80% criterion (Figure S2). Female mice showed age‐related declines in spatial working memory, as assessed by cognitive flexibility and maximal learning in the reversal phase (Figure S2). However, the female cohort could not be statistically stratified based on the lack of variability observed in entries required during the reversal using the metric of the 80% criterion. This discrepancy between sexes may reflect reduced sensitivity of the 80% criterion in detecting differences or an inherent lack of cognitive heterogeneity within the female population at this age. Given the inability to stratify aged females and the robust variability observed in aged males, our subsequent analyses focused on the cognitively stratified aged males to investigate mechanisms underlying age‐related cognitive heterogeneity.

### 2.2 Hippocampal Reactive Gliosis Delineates Cognitive Status in Aging

**FIGURE 2:**
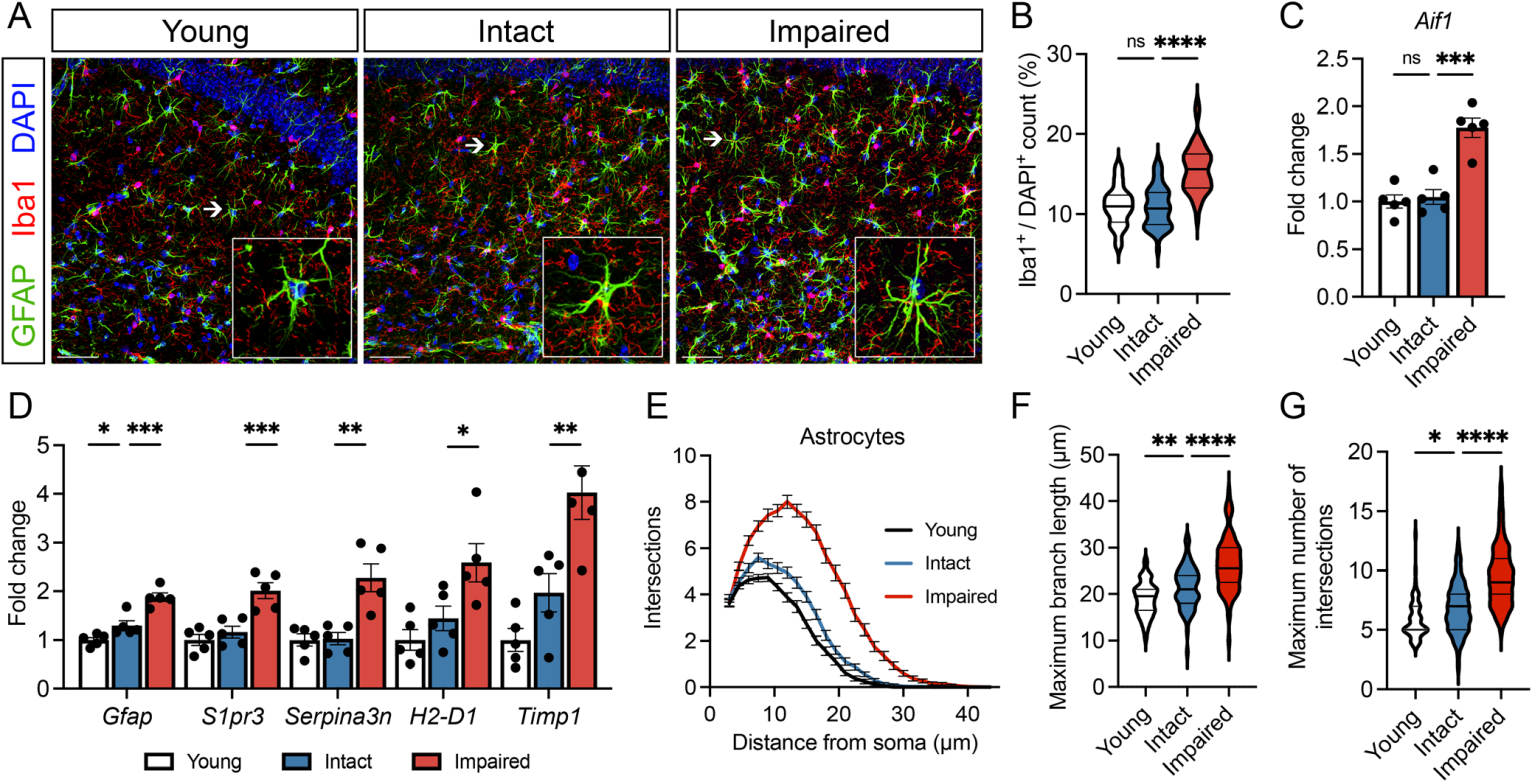
Cognitively impaired aged male mice show increased markers of reactive gliosis within the hippocampus. (A) Representative confocal images of immunostained hippocampal sections within the CA1 region labeled for GFAP (green; astrocytes), Iba1 (red; microglia), and DAPI (blue; nuclei) from cognitively stratified mice. White arrows specify the cells depicted in each insert. Scale bar, 50 μm. (B) Proportion of Iba1+ cell number normalized to total cell number (determined via DAPI^+^ nuclei count) in the CA1 region of the hippocampus. 3–4 sections were analyzed per *n*. (C) Transcriptional expression of the microglial marker *Aif1* in the hippocampus. *n* = 5 mice/group. (D) Transcriptional expression of the astrocyte cytoskeletal element *Gfap* and astrocyte reactivity markers (*S1pr3, Serpina3n, H2‐D1, Timp1*) within the hippocampus. *n* = 5 mice/group. (E) Quantification of the number of GFAP^+^ astrocyte projections plotted against the radial distance from the soma obtained via Sholl analysis in the CA1 region of the hippocampus. (F, G) Violin plots depicting maximal branch length and maximal number of intersections of GFAP+ astrocytes assessed by Sholl analysis. For graphs B–G, colors represent the following: Young (black), aged intact (blue), aged impaired (red). Error bars: Mean ± SEM. *p < 0.05, **p < 0.01, ***p < 0.001, ****p < 0.0001.

Reactive gliosis, characterized by morphological and functional changes in microglia and astrocytes, is closely associated with neuroinflammation and is thought to significantly contribute to age‐related dementias (De Sousa 2022). To investigate the role of reactive gliosis in age‐related cognitive heterogeneity, we analyzed high‐magnification confocal images of Iba1‐ and GFAP‐immunolabeled (markers of microglia and astrocytes, respectively) cells within the hippocampal CA1 region (Figure 2A). We also assessed the transcriptional expression of genes associated with reactive gliosis (Jurga et al. 2020; Liddelow et al. 2017) in isolated hippocampi from cognitively stratified aged male mice via quantitative real‐time PCR (qPCR).

We observed a significant increase in the number of Iba1‐immunolabeled microglia specifically in the cognitively impaired subgroup (Figure 2B). This increase was accompanied by elevated transcriptional expression of *Aif1* in impaired mice, the gene encoding Iba1 prominently expressed in microglia (Figure 2C). As microglial reactivity is closely linked with cellular proliferation and elevated *Aif1* expression (Jurga et al. 2020), these findings suggest an upregulation of microglial activation that is uniquely associated with cognitive impairment in aging.

We then examined astrocyte reactivity within the cognitive stratified subgroups through transcriptional profiling of established astrocyte reactivity markers implicated in neurological diseases (Escartin et al. 2021) and morphological assessment via Sholl analysis. Cognitively impaired males displayed significantly higher expression of *Gfap*, a key cytoskeletal component in astrocytes, compared to their cognitively intact counterparts (Figure 2D). Additionally, the expression of *Serpina3n*, *S1pr3*, *H2‐D1*, and *Timp1* (Figure 2D), markers of reactive astrogliosis previously implicated in aged animals to be induced by activated microglia (Clarke et al. 2018), was elevated in cognitively impaired mice. Morphological analysis of individual astrocytes through Sholl analysis (Figure 2E) revealed that, in line with modest age‐related increases in *Gfap* expression, hippocampal CA1 astrocytes of cognitively intact aged mice displayed moderate increases in maximal branch length and maximal number of intersections (Figure 2F,G). However, these morphological changes were significantly more pronounced in the cognitively impaired subgroup (Figure 2F,G). The total number of GFAP‐immunolabeled astrocytes did not differ significantly between cognitively intact and impaired animals (Figure S3A), suggesting a nonproliferative reactive astrogliosis in the hippocampus of impaired mice.

Overall, these data indicate that a neuroinflammatory phenotype characterized by heightened reactive gliosis, encompassing both microglial activation and astrocyte reactivity within the hippocampus, is a distinct feature distinguishing cognitive impairment from resilience.

### 2.3 Cognitively Impaired Aged Mice Exhibit Increased Markers of Cellular Senescence in the Hippocampus

Cellular senescence, a hallmark of biological aging (López‐Otín et al. 2013), is a major driver of systemic inflammation, including neuroinflammation. Given the neuroinflammatory phenotype marked by reactive gliosis in the hippocampus of cognitively impaired aged male mice, we assessed markers of hippocampal cellular senescence via qPCR and histological approaches.

**FIGURE 3:**
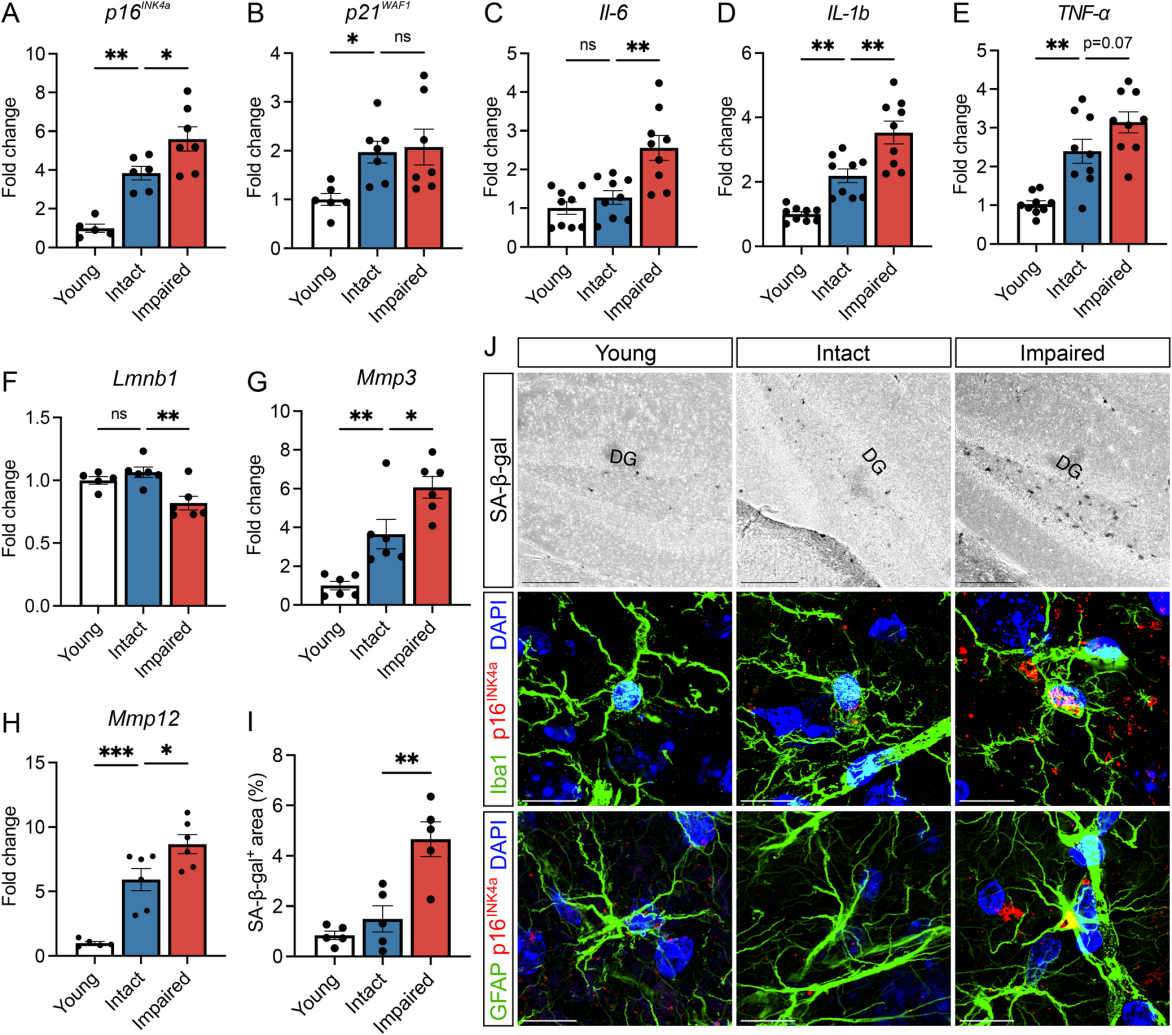
Cognitively impaired aged male mice display increased signatures of cellular senescence within the hippocampus. (A–H) Transcriptional expression of cell cycle arrest markers (*p16* ^*INK4a*^ and *p21* ^*WAF1*^) and senescence‐associated markers, including secreted cytokines (*Il‐6*, *Il‐1b*, *Tnf‐ɑ*), nuclear lamin component (*Lmnb1*), and matrix metalloproteases (*Mmp3*, *Mmp12*) within the hippocampus. *n* = 5–9 animals/group. (I) Quantification of senescence‐associated beta‐galactosidase (SA‐β‐gal) staining within the hippocampus (representative images, panel J; quantification from 3 to 4 sections per animal, *n* = 5 animals/group). (J) Representative sagittal images of senescence‐associated beta‐galactosidase (SA‐β‐gal) and p16^INK4a^ immunolabeling co‐localizing with microglial and astrocyte markers Iba1 and GFAP within the dentate gyrus of the hippocampus. Scale bars, 200 μm (SA‐β‐gal), 10 μm (p16INK4a). DG denotes the granule cell layer of the dentate gyrus. For graphs A–I, colors represent the following: Young (black), aged intact (blue), aged impaired (red). Error bars: Mean ± SEM. *p < 0.05, **p < 0.01, ***p < 0.001.

With respect to markers of cell cycle arrest, we observed significant increases in the expression of *p16* ^*INK4a*^ (*Cdkn2a*), a cyclin‐dependent kinase inhibitor commonly utilized as a marker of senescence, both with age and cognitive status (Figure 3A). In contrast, the expression of the senescence‐associated *Cdkn1a* transcript variant 2 (*p21* ^*WAF1*^) (López‐Domínguez et al. 2021) was increased with age but remained unchanged by cognitive status (Figure 3B) in male mice. We next evaluated components of the senescence‐associated secretory phenotype (SASP), including the expression of pro‐inflammatory cytokines and matrix‐degrading enzymes. While *TNF‐*α expression showed a trending increase (*p* = 0.07), *Il‐6* and *Il‐1b* were significantly upregulated in cognitively impaired mice compared to cognitively intact counterparts (Figure 3C–E). Similarly, impaired mice showed downregulation of the nuclear lamin component *Lmnb1* and increases in matrix metalloprotease (*Mmp3*, *Mmp12*) expression in comparison to aged, cognitively intact mice (Figure 3F–H).

In addition to increased expression of cell cycle arrest machinery and pro‐inflammatory factors, senescent cells display increased lysosomal activity detectable through senescence‐associated β‐galactosidase (SA‐β‐gal) staining (Ogrodnik et al. 2024). We performed SA‐β‐gal staining and identified an increased relative area of staining in the hippocampus, most notably within the dentate gyrus, of cognitively impaired aged mice (Figure 3I,J). To identify the cell types exhibiting SA‐β‐gal activity, we conducted double‐labeling experiments combining SA‐β‐gal staining with immunolabeling. These experiments identified co‐localization of SA‐β‐gal activity with Iba1 and GFAP, markers for microglia and astrocytes, respectively (Figure S4). Moreover, immunofluorescent labeling of p16^INK4a^ co‐localized with Iba1 and GFAP within the dentate gyrus of impaired males (Figure 3J and Figures S5 and S6), further supporting the association of glial cell senescence with impairment.

Given the observed age‐ and impairment‐related increases in gliosis and senescence markers, we performed linear regressions of cognitive performance metrics from the PhenoTyper against the transcriptional expression of these factors. Reactive gliosis markers (e.g., *Gfap*, *Aif1*) were negatively correlated with cognitive flexibility and positively associated with the number of entries needed to reach the criterion during the reversal phase (Table S1). Similarly, senescence markers, including *p16* ^*INK4a*^ and SASP factors such as *Il‐6*, showed significant correlations with these cognitive metrics (Table S1). Notably, *p16* ^*INK4a*^ expression also correlated positively with markers of reactive gliosis (*Gfap* and *Aif1*) in addition to SASP factors (*Il‐6* and *TNF‐*α) (Table S1).

Both senescent microglia and disease‐associated microglia (DAM), an activated microglial phenotype linked to neurodegenerative diseases including AD, are known to secrete pro‐inflammatory cytokines that induce reactive astrogliosis (Liddelow et al. 2017). Additionally, recent studies have highlighted overlaps between reactive astrogliosis and senescent astrocyte phenotypes (Cohen and Torres 2019). To investigate whether chronic activation of reactive astrogliosis alone could drive a senescent phenotype, we treated primary astrocyte cultures with a cytokine cocktail (TNF‐α, IL‐1a, and C1q) previously shown to induce reactive astrogliosis and astrocytic secretion of proinflammatory cytokines (e.g., IL‐1b, TNF‐α) (Liddelow et al. 2017).

Following cytokine treatment, we confirmed the induction of reactive astrogliosis through increased expression of known reactivity markers (*S1pr3, C3, Serpina3n, H2‐D1, Timp1, Cxcl10, Lcn2*) and downregulation of the water channel *Aqp4* (Figure S7A). Furthermore, the cytokine‐treated astrocytes exhibited increased expression of SASP‐associated pro‐inflammatory cytokines (*Il‐6*, *Il‐1b*, *TNF‐*α), the chemokine *Ccl2*, and the matrix metalloprotease *Mmp3* (Figure S7B). Importantly, we also observed significant upregulation of *Cdkn2a* (encoding cell cycle arrest protein p16^INK4a^) in cytokine‐treated astrocytes, with no changes in *Cdkn1a* expression (Figure S7B). These findings suggest that reactive astrogliosis can promote a senescence‐like phenotype in astrocytes or contribute to the paracrine induction of senescence in neighboring cells (Acosta et al. 2013) through its pro‐inflammatory secretome.

In summary, these data suggest a strong interplay between cellular senescence and reactive gliosis in the hippocampus, with differential expression of these phenotypes potentially driving disparities in cognitive function with age.

### 2.4 Senolytic Treatment Ameliorates Cognitive Heterogeneity and Impairment‐Associated Increases in Pro‐Inflammatory Gene Expression in Aged Males

Given the association between the accumulation of senescence markers and cognitive status with age, we investigated the effects of the senolytic regimen, dasatinib and quercetin (D + Q) (Zhang et al. 2022), on age‐related cognitive heterogeneity. Aged male and female mice received D + Q or vehicle via oral gavage starting at 22 months of age, administered for three consecutive days every 2 weeks over an 8‐week period.

During the study, 2 of 28 vehicle‐treated and 3 of 25 senolytic‐treated aged males reached humane endpoints before cognitive assessment and were excluded from analyses. At the conclusion of treatment at 24 months of age, both groups exhibited a modest (4%) weight reduction compared to pretreatment weights (Figure S8A), with no significant differences between treatment groups at either time point. To assess whether senolytic treatment influenced physical characteristics of aging, we conducted body composition and frailty assessments. While age‐related increases in frailty scores were noted, lean/fat mass distribution and frailty scores did not differ between vehicle‐ and senolytic‐treated aged mice (Figure S8B–I).

**FIGURE 4:**
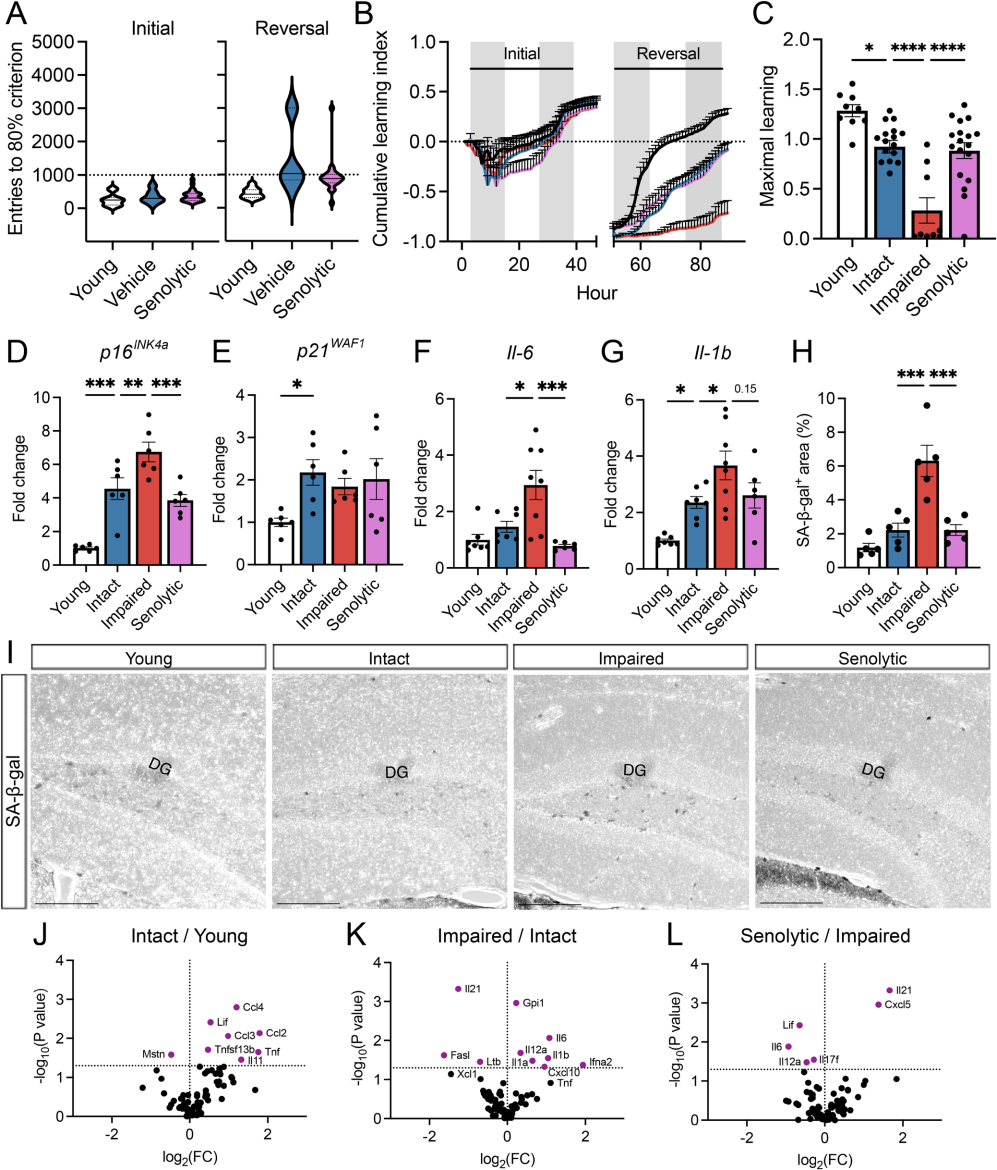
Senolytic treatment ameliorates cognitive heterogeneity and hippocampal senescence markers in aged male mice. (A) Entries needed to reach 80% criterion in the initial and reversal learning phases of young, vehicle‐ and senolytic‐treated male mice. (B) Cumulative learning index depicts separations between vehicle‐treated cognitively impaired mice and vehicle‐treated cognitively intact mice and senolytic‐treated mice during the reversal phase. (C) Maximal learning of aged cognitively impaired mice at the end of the reversal phase was significantly decreased compared to vehicle‐treated cognitively intact and senolytic‐treated mice. (D–G) Transcriptional expression of cell cycle arrest markers (*p16* ^*INK4a*^, *p21* ^*WAF1*^) and SASP factors (*IL‐6*, *IL‐1b*) within the hippocampus of vehicle‐treated cognitively stratified and senolytic‐treated aged male mice. (H, I) Quantification and representative images of senescence‐associated beta‐galactosidase staining within the hippocampus. DG denotes the granule cell layer of the dentate gyrus. Scale bar, 200 μm; 3–4 sections per animal, *n* = 5 mice/group. (J–L) Volcano plots depicting the fold change of cytokines and chemokines assessed via the RT^2^ profiler between (J) vehicle‐treated intact mice and young, (K) vehicle‐treated impaired and intact mice, and (L) senolytic‐treated and vehicle‐treated impaired aged mice. Points above the dotted line denote significantly different genes (*p* < 0.05). For all histograms, colors represent the following: Young (black), vehicle‐treated intact (blue), vehicle‐treated impaired (red), and senolytic‐treated (pink). Shaded bars in graphs B represent the dark periods of the L:D cycle. Error bars: Mean ± SEM. *p < 0.05, ***p < 0.001, ****p < 0.0001.

Cognitive assessment at 24 months of age revealed no significant differences in circadian activity between treatment groups based on the onset of activity during the dark phase (Figure S9). Both young and aged mice successfully learned the initial task regardless of treatment (Figure 4A). However, as in our initial cohorts, vehicle‐treated aged mice displayed substantial heterogeneity in reversal learning performance (Figure 4A). In contrast, most senolytic‐treated males performed comparably to aged cognitively resilient mice, exhibiting significantly higher maximal learning compared to cognitively impaired vehicle‐treated counterparts (Figure 4B,C). In female aged mice, senolytic treatment had no effect on circadian activity compared to vehicle‐treated controls (Figure S10). Cognitive performance was similarly unaffected, with no significant differences in entries to reach the criterion or maximal learning during reversal learning (Figure S10).

To validate the effect of senolytic treatment, we assessed senescence markers in the hippocampus and their association with cognitive status. In vehicle‐treated cognitively intact impaired subgroups, expression patterns of cell cycle arrest markers (*p16* ^*INK4a*^ and *p21* ^*WAF1*^), SASP factors *Il‐6* and *Il‐1b*, and SA‐β‐gal staining mirrored those observed in our initial untreated, stratified cohorts (Figure 4D–I). Compared to vehicle‐treated cognitively impaired aged animals, senolytic‐treated males exhibited significantly reduced levels of *p16* ^*INK4a*^ and *Il‐6*, with a decreasing trend in *Il‐1b* (*p* = 0.15). No significant changes were observed in *p21* ^*WAF1*^ expression across aged male treatment groups. Notably, in females, *p21* ^*WAF1*^ was the only senescence marker elevated in the aged vehicle‐treated group that was attenuated with D + Q (Figure S10J–N).

To obtain a comprehensive transcriptional profile of key cytokines and chemokines associated with cognitive statuses and markers of impairment amenable to senolytic therapy, we utilized a multiplex qPCR assay (RT^2^ profiler, QIAGEN) targeting 86 inflammation‐associated genes (Figure S11A). Among the most differentially expressed genes, aged mice exhibited increased expression of chemoattractants (*Ccl2, Ccl3, Ccl4*) and cytokines (*TNF*, *Tnfsf13b, Il‐11*) while *Mstn*, a gene implicated in synapse function and neuronal growth, was downregulated (Figure 4J). We observed upregulation of multiple pro‐inflammatory cytokines (*Il‐6, Il‐1a, Il‐1b, Il‐12a, Ifna2*) and the chemotactic factor *Cxcl10*, with downregulations in *Il‐21*, *Fasl*, and *Ltb* expression specific to impairment (Figure 4K). Senolytic treatment significantly reduced *Il‐6, Il‐12a, Il‐17f*, and *Lif* expression relative to vehicle‐treated aged‐impaired mice (Figure 4L). These findings suggest Il‐6 signaling may be a key driver of differential cognitive trajectories and a target amenable to senolytic therapy.

To further characterize the molecular pathways underlying differences in cognitive status and senolytic response, we performed Ingenuity Pathway Analysis (IPA) using our cytokine and chemokine expression profiles (Figure S11B–D). Comparisons between cognitively intact and impaired mice revealed upregulation of pathways associated with senescence, T‐cell receptor signaling, acute phase response signaling, Th17 activation, and IL‐23 signaling, while IL‐27 signaling, NK cell signaling, and NFKBIE signaling pathways were downregulated (Figure S11C). In senolytic‐treated animals, we observed downregulation in senescence‐ and Th1‐associated pathways, with concomitant upregulation of Th2 and anti‐inflammatory IL‐10 signaling pathways relative to impaired mice (Figure S11D). Collectively, these results highlight distinct inflammatory profiles associated with cognitive status and provide further evidence of senescence pathways contributing to cognitive impairment.

### 2.5 Senolytic Treatment Mitigates Hippocampal Reactive Gliosis and Deficits in Mitochondrial Respiration With Cognitive Impairment

**FIGURE 5:**
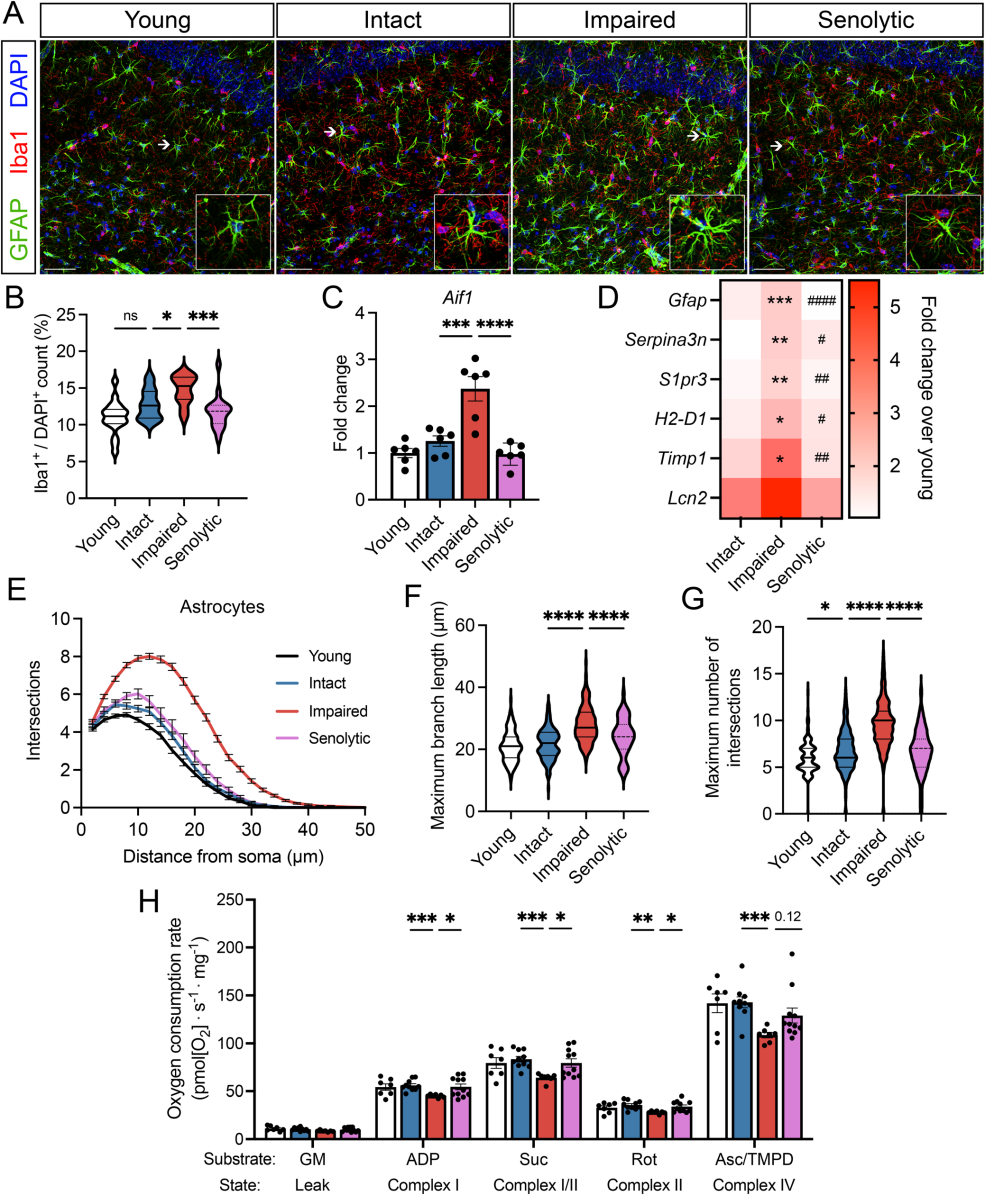
Reactive gliosis and deficits in mitochondrial respiration in the hippocampus associated with cognitive impairment are ameliorated by senolytic therapy. (A) Representative confocal images of immunostained hippocampal sections within the CA1 region labeled for GFAP (green; astrocytes), Iba1 (red; microglia), and DAPI (blue; nuclei) from young, vehicle‐treated, cognitively stratified, and senolytic‐treated male mice. White arrows specify the cells depicted in each insert. *n = 5* animals/group. Scale bar, 50 μm. (B) Proportion of Iba1+ cell number normalized to total cell number (determined via DAPI^+^ nuclei count) in the CA1 region of the hippocampus. 3–4 sections were analyzed per *n*. (C) Transcriptional expression of the microglial marker *Aif1* of the hippocampus of young, vehicle‐ and senolytic‐treated aged male mice. *n* = 6 mice/group. (D) Expression heatmap of reactive astrocyte‐associated genes (expressed as fold change over young), including *Gfap*, *Serpina3n*, *S1pr3*, *H2‐D1*, and *Lcn2*. * and # denote significance between intact/impaired and impaired/senolytic‐treated groups, respectively. *n* = 6 animals/group. (E) Quantification of the number of astrocyte projections plotted against the radial distance from the soma obtained via Sholl analysis. (F, G) Maximal branch length and maximal number of intersections of GFAP+ astrocytes were quantified using Sholl analysis. (H) Oxygen consumption rate in permeabilized hippocampi following sequential addition of the substrates glutamate/malate (GM), ADP (2.5 mM), succinate (Suc), rotenone (Rot), and ascorbate/TMPD (Asc/TMPD). *n* = 7–11 animals/group. For graphs B, C and E–H, colors represent the following: Young (black), vehicle‐treated aged intact (blue), vehicle‐treated aged impaired (red), senolytic‐treated (pink). Error bars: Mean ± SEM. *p < 0.05, **p < 0.01, ***p < 0.001, ****p < 0.0001.

Given the positive association of senescence and reactive gliosis and the beneficial effects of senolytic treatment on cognitive trajectories, we investigated whether hippocampal microglial and astrocytic reactivity was mitigated by senolytics (Figure 5A). In comparison to vehicle‐treated cognitively impaired mice, senolytic treatment attenuated the increases in microglial cell number and *Aif1* expression in the hippocampus (Figure 5B,C). Notably, the upregulation of reactive astrocyte genes associated with impairment (*Gfap*, *Serpina3n*, *S1pr3*, *H2‐D1*, and *Timp1*) was markedly decreased in the senolytic‐treated group (Figure 5D). Similarly, vehicle‐treated cognitively impaired aged animals exhibited greater astrocyte arborization, characterized by longer maximal branch lengths and increased numbers of intersections (Figure 5E–G). In contrast, senolytic treatment had minimal impact on gliosis‐related markers in aged females (Figure S10F–I), suggesting sex‐specific profiles of gliosis amenable to senolytic therapy. Taken together, these data suggest that senolytic therapy mitigates variability in age‐related cognitive deficits and reduces impairment‐associated hippocampal gliosis in male mice.

Senescence is thought to drive metabolic disturbances in aging. We have previously reported that cognitively impaired aged male mice exhibit downregulation in mitochondrial OXPHOS genes and reduced mitochondrial function in the hippocampus (Logan et al. 2023). We therefore assessed the impact of senolytic treatment on hippocampal mitochondrial function via oxygen consumption rates in permeabilized hippocampi from vehicle‐ and senolytic‐treated aged male mice using high‐resolution respirometry (Figure 5H). No significant age‐ or treatment‐related changes were observed in leak‐state respiration following glutamate/malate (GM) addition. Oxygen consumption rates in young and cognitively intact aged mice were comparable across all substrates. However, cognitively impaired aged mice exhibited reduced complex I‐, II‐, and IV‐stimulated respiration relative to cognitively intact aged mice. Notably, these deficits were absent in senolytic‐treated animals, whose oxygen consumption rates were similar to those of young and cognitively intact aged mice. These findings suggest that the constellation of changes resulting from senolytic treatment facilitated optimal hippocampal mitochondrial function, putatively supporting cognitive resiliency.

## 3 Discussion

The progressive loss of cognitive function and increase in the incidence of dementia with advancing age lead to substantial declines in quality of life, heightened dependency, and significant socio‐economic burden. Importantly, not all individuals will experience cognitive decline as they age. This observation suggests that specific cellular and molecular alterations in the aging brain may shape cognitive trajectories and underpin variability in cognitive function.

A major impediment to elucidating these mechanisms in preclinical studies has been the limited sensitivity of classical cognitive testing paradigms (e.g., RAWM, novel object recognition [NOR], etc.) to detect subtle differences in cognition (Logan et al. 2018). These assays are susceptible to multiple sources of confounds, including protocol discrepancies between and within labs, environmental conditions, and experimenter handling. Moreover, sensory and motivational differences across aging are also well recognized to confound behavioral tests, including NOR and Y‐maze performance (Gerlai 2001). Many protocols also test mice during the light phase of the light/dark cycle, a period when rodents are less active. Even when reversed day/light paradigms are implemented, the limited number of trials that can pragmatically be conducted by experimenters limits the utility of these protocols in detecting nuanced cognitive differences within aged animals. This is particularly true in mice exhibiting perseveration behavior, potentially requiring tens or hundreds of trials to exhibit effective learning. In this study, we leveraged a robust, minimally stressful testing procedure to detect age‐related changes in cognitive function (Logan et al. 2018, 2019) and showed to reliably differentiate cognitive performance variables in C57Bl/6N mice (Logan et al. 2023). As a result, these analyses improve our understanding of the molecular landscape that specifically defines cognitive impairment in aging.

Human cognitive aging spans a broad spectrum—while some individuals retain cognitive abilities well into old age, others exhibit marked declines. Hippocampal‐dependent spatial working memory loss is one of the earliest cognitive impairments in AD and manifests as diminished capacity for discrimination learning in both human and mouse models (Remmelink, Smit, et al. 2016). Reversal learning, which is heavily reliant on working memory (Klanker et al. 2013), is notably impacted in aging. Compromised executive functions, such as problem‐solving and decision‐making, often decline with age, with deficits hypothesized to stem from impairments in working memory (Corbo et al. 2025; Idowu and Szameitat 2023). The PhenoTyper's CognitionWall tests reversal learning by switching reward delivery based on successful entry into three different points, a task that is particularly challenging for older subjects. Paralleling cognition in humans requiring more time to change strategy in comparison to initial learning (Dias et al. 1997; Tsuchida et al. 2010), mice require more entries to reach criterion during reversal learning (Remmelink, Smit, et al. 2016). Moreover, mice make increased perseverative errors compared to neutral errors during reversal learning (Remmelink, Smit, et al. 2016), akin to humans undergoing reversal learning tasks (den Ouden et al. 2013). These behaviors are mediated by the orbitofrontal cortex (OFC), a key region coordinating flexible stimulus‐reinforcement learning (Hornak et al. 2004; Tsuchida et al. 2010). Importantly, OFC lesions in mice impair reversal learning in CognitionWall tasks while leaving discrimination learning and general activity intact (Remmelink, Smit, et al. 2016). These findings support the translational validity of the PhenoTyper paradigm in assessing age‐related cognitive impairments and suggest strong parallels between rodent and human cognitive flexibility in aging.

Aging is known to disrupt functional connectivity between brain regions critical for learning and memory, including the hippocampus and prefrontal cortex (Ranjbar‐Slamloo et al. 2025). Despite these disruptions, some studies suggest that aged individuals with intact cognitive abilities may compensate by recruiting alternative networks or enhancing connectivity in certain regions. For example, increased positive functional connectivity between the hippocampus and dorsolateral prefrontal or parietal regions has been associated with better memory performance in aged humans (Grady et al. 2003). Conversely, the disconnect between the hippocampus and prefrontal cortex is associated with cognitive decline in Alzheimer's disease (Grady et al. 2001). It is also important to note that higher cognitive reserve is associated with sustained cognitive abilities with age in humans (Barulli et al. 2013). Importantly, individuals noted as “super‐agers” are shown to harbor comparable cognitive performance to young individuals [reviewed by: (Glisky 2007; Powell et al. 2023; Zanto and Gazzaley 2019)]. Thus, our studies to cognitively stratify aged mice are translationally relevant and instrumental in understanding the mechanisms that underlie disparity in cognition akin to those seen in humans.

Over the past decade, the significance of cellular senescence in brain aging has grown substantially. Studies using transgenic mouse models (e.g., INK‐ATTAC, p16‐3MR) and senolytic agents (e.g., D + Q, navitoclax) have demonstrated that targeting senescent cells can mitigate numerous age‐ and disease‐related brain pathologies. Of particular relevance to our work, senolytic interventions have shown promise in improving cognitive abilities in aging (Budamagunta et al. 2023; Ogrodnik et al. 2021) and AD (Bussian et al. 2018; Zhang et al. 2019) models, with ongoing clinical trials exploring their therapeutic potential (Gonzales et al. 2023). Building upon these insights, our data reveal that aged male mice with pronounced spatial working memory deficits exhibited increased hippocampal reactive gliosis and markers of cellular senescence compared to cognitively intact aged mice. Moreover, senolytic treatment alleviated age‐related cognitive heterogeneity, with mice receiving D + Q displaying preserved spatial working memory and reduced signatures of senescence and gliosis, comparable to cognitively intact aged animals. Extending our previous finding that hippocampal mitochondrial dysfunction aligns with cognitive impairment (Logan et al. 2023), we show that senolytic therapy promoted complex‐specific mitochondrial function comparable to cognitively intact mice. Although it remains unclear whether these functional improvements precede or result from the accumulation of senescent cells, our findings suggest that senescence is a critical factor shaping divergent cognitive outcomes with age. As such, future investigation into the mechanisms driving differential senescence burdens across individuals is critical to understanding the genesis of senescence with age.

The neuroinflammatory milieu that drives reactive astrogliosis (Liddelow et al. 2017) is thought to contribute to neurological deficits in physiological aging (Clarke et al. 2018). In our study, we show that markers linked to proinflammatory neurotoxic astrocyte phenotypes (e.g., *S1pr3*, *Serpina3n*, *H2‐D1*) (Escartin et al. 2021) were elevated specifically in cognitively impaired mice. This shift may be fueled, at least in part, by increased microglial activation in the impaired group that is attenuated with senolytic therapy, suggesting a potential mechanistic target for future investigation in age‐related cognitive heterogeneity. In support, our in vitro studies show that induction of reactive astrocytes using a cocktail of microglial‐secreted cytokine effectors promoted a senescent profile of astrocytes that largely recapitulated the molecular phenotype observed in vivo with cognitive impairment. In addition to senescent glia, cytokine release from T cells within the CNS has been implicated in modulating neuronal and glial function in aging and neurodegenerative diseases (Altendorfer et al. 2022; Machhi et al. 2021). Our IPA analyses highlight possible differences in T cell signaling pathways with cognitive status. Interestingly, we found an upregulation of the Th17 activation pathway in our cognitively impaired subgroup. Th17‐derived factors have been shown to induce reactive astrocytes with proinflammatory phenotypes, contributing to further recruitment of microgl and additional T cells across the blood–brain barrier (Prajeeth et al. 2017).

While numerous reports detail senescence in microglia (Bussian et al. 2018; Matsudaira et al. 2023; Ogrodnik et al. 2021; Rachmian et al. 2024) and oligodendrocyte‐lineage (Zhang et al. 2019) cells, emerging evidence suggests astrocyte senescence, a fate occurring in addition to reactive astrogliosis, may be a distinct contributor to cognitive aging (Lye et al. 2019; Suda et al. 2021) and neurodegenerative diseases (Chinta et al. 2018; Cohen and Torres 2019). Neuroinflammatory astrocytes in the hypothalamus of aged mice have been reported to express senescence markers such as p16^INK4a^ (Suda et al. 2021), and the accumulation of senescent astrocytes has been linked to cognitive decline in humans (Lye et al. 2019). In our data, we observed colocalization of p16^INK4a^ and SA‐β‐gal with microglia and astrocyte markers, suggestive that glial cells constitute a key senescent population in the impaired brain. However, we should emphasize that we have not ruled out the possibility of senescence in other brain cell types (e.g., oligodendrocyte lineages, neurons) or peripheral immune cells such as T cells. Although neurons may exhibit senescence‐like phenotypes under certain conditions (Jurk et al. 2012), the concept of neuronal senescence remains highly debated in normative aging, primarily because classical markers and definitions of senescence are challenging to apply to post‐mitotic cells (Dos Melo Santos et al. 2024). Evidence suggests that in vivo neuronal senescence‐like phenotypes occur more frequently in pathological contexts, such as tauopathy (Dehkordi et al. 2021) or induced neurons from AD patients (Herdy et al. 2022). The incidence of neuronal senescence in healthy aging in the absence of neurodegenerative pathology or stressors (e.g., high fat diet) remains less clear [reviewed by: (Dos Melo Santos et al. 2024)]. In mice, senescence‐like neuronal phenotypes can be p21‐dependent (Jurk et al. 2012), yet our data show no significant differences in *p21* ^*WAF1*^ expression between the cognitive status of aged male mice. As the senescence field moves toward improved consensus on defining markers of senescence, higher‐resolution approaches such as single‐cell RNA sequencing or spatial transcriptomics will be crucial to pinpoint the specific cell populations undergoing senescence in cognitive impairment and to understand how these mechanisms vary across genetically diverse backgrounds.

Although cognitive performance likely spans a continuum in aged populations, our analyses focused on divergent cognitive status to explore molecular correlates of impairment rather than the effect of age alone. At present, we are unable to delineate a timeline for the trajectory of cognitive decline due to technical limitations of repeated cognitive testing in mice, including carryover effects and the risk of overtraining (Cnops et al. 2022). Furthermore, another limitation of the present study was the inability to cognitively stratify aged female mice and thereby differentiate sex‐specific effects of senescence profiles on cognition. Based on our current analyses, female mice at 24 months do not appear to exhibit the same heterogeneity as males at this age, with senolytic treatment showing little effect on spatial working memory deficits in female mice. Notably, previous studies using APP models of AD have implicated sexually dimorphic effects of senolytics on cognition (Fang et al. 2024), further suggestive of critical differences in senescence‐driven processes between sexes. Together, these findings raise key questions about sex‐specific differences in the induction of senescence, senescence burden, and profile of senescent cells (e.g., SASP) in driving heterogeneity during normative aging in females that warrant further investigation. Finally, the scope of our work was confined only to the hippocampus, given its pivotal role in spatial learning and memory. However, it is likely that other brain regions and peripheral effectors, as described above, play important roles in the differences in cognition observed, which will need further exploration.

In conclusion, our findings highlight a unique intersection of reactive gliosis and cellular senescence as key determinants of cognitive heterogeneity in aged male mice. By employing a high‐resolution cognitive testing platform and examining aged animals by cognitive status, we demonstrate that senolytic therapy effectively reduces reactive gliosis and senescence burden associated with cognitive impairment. This work supports the targeting of senescent cells as a promising strategy in mitigating age‐related cognitive impairment and highlights avenues for future investigation toward unraveling the cellular and regional contributions that govern resilience and vulnerability in the aging brain.

## 4 Methods

### 4.1 Animals

All animal procedures were approved and conducted in accordance with the guidelines of the Institutional Animal Care and Use Committee at the University of Oklahoma Health Sciences (OUHS). Aged male and female C57BL/6N mice were obtained from the NIA colony at Charles River. Mice were housed (2–4 per cage) in conventional rodent facilities at OUHSC in Allentown XJ cages with Anderson's Enrich‐o‐cobb bedding. Mice were maintained in a 12‐h light/12‐h dark cycle at 21°C with *ad libitum* access to standard irradiated bacteria‐free rodent chow (5053 Pico Lab, Purina Mills, Richmond, IN) and reverse osmosis filtered water. Animals were intracardially perfused with 0.1 M phosphate‐buffered saline (1X PBS; pH 7.4) containing heparin (2 units/mL) prior to molecular and immunohistochemical analyses. In all experiments, male and female mice were assessed and analyzed separately due to the varied timeline for procurement of cohorts from NIH.

### 4.2 Automated Home‐Cage Spatial Memory Assessment (PhenoTyper)

Evaluation of spatial working memory was performed using an automated home‐cage testing paradigm (PhenoTyper) as previously described (Logan et al. 2023, 2018). Following acclimatization to the home‐cage, the spontaneous activity of individual mice was assessed over 90 h, with movement recorded via an infrared‐sensitive video camera using EthoVision XT 14 software. During the initial discrimination learning phase (hours 1–49), mice were required to pass through the left entrance of the Cognition Wall to receive a food reward on a fixed ratio (five correct entries/reward) schedule. Reversal learning was assessed during hours 51–90, requiring mice to enter the right entrance of the cognition wall. Success rates for both acquisition and reversal learning were calculated and defined as the percentage of correct entries of the trailing 30 entries needed to reach an 80% success rate (efficiency criterion). Aged mice were then cognitively stratified based on the number of entries to reach criterion during the reversal phase using young performance metrics (1000 entries) defined in our previous publication (Logan et al. 2023). The independent learning index was calculated per hour based on correct entries minus incorrect entries divided by the total number of entries and plotted as a cumulative learning index (CLI) for acquisition and reversal learning. The CLI is a visual representation of compounded learning over time, which accounts for the total entries made by each mouse, irrespective of whether they met efficiency criteria. Additionally, cognitive flexibility and maximal learning were calculated as correct entries minus incorrect entries divided by the total number of entries during the first dark phase of reversal learning and over the reversal period, respectively.

### 4.3 Quantitative PCR

Total hippocampal RNA was extracted using the RNeasy Mini kit (Qiagen) with cDNA prepared from equal concentrations of total RNA (1000 ng) using the High‐Capacity RNA‐to‐cDNA kit (Applied Biosystems). qRT‐PCR was performed using the following gene‐specific TaqMan expression assays (Applied Biosystems): *p16* ^*INK4a*^ (forward: 5′‐AACTCTTTCGGTCGTACCCC‐3′, reverse: 5′‐TCCTCGCAGTTCGAATCTG‐3′), *p21* ^*WAF1*^ (transcript variant 2; APT2FJW), *Il‐6* (Mm00446190_m1), *Il‐1b* (Mm00434228_m1), *TNF‐ɑ* (Mm00443258_m1), *Mmp3* (Mm00440295_m1), *Mmp12* (Mm00440295_m1), *Lmnb1* (Mm00521949_m1), *Gfap* (Mm01253033_m1), *Aif1* (Mm00479862_g1), *S1pr3* (Mm02620181_s1), *Serpina3n* (Mm00776439_m1), *H2‐D1* (Mm04208017_mH), *Timp1* (Mm01341361_m1), *Lcn2* (Mm01324470_m1). *Cytochrome c‐1* (Mm00470540_m1) and *Ube2d2* (Mm00785931_s1) were used as housekeeping genes for normalization. Quantitative PCR and melt‐curve analyses were performed using TaqMan Universal PCR Master Mix with UNG (Applied Biosystems) and the QuantStudio 12K Flex System (Applied Biosystems). Expression data for each independent sample were calculated from two replicates normalized to the expression of the geometric mean of housekeeping genes using the comparative C_T_ method. Data presented are fold changes over mean young (or control for in vitro studies) expression.

### 4.4 Immunofluorescence and Histological Analyses

Standard immunofluorescent labeling was performed as previously described (Baier et al. 2022). Briefly, brains were harvested following perfusion and fixed in 4% paraformaldehyde in 1X PBS overnight at 4°C. Fixed brains were then cryoprotected in 30% sucrose in PBS, embedded in Tissue‐Tek OCT compound (Sakura), and frozen at −80°C. Sagittal sections (30 μm) were obtained using a cryostat and stored in cryopreservative solution (30% glycerol, 30% ethylene glycol, 40% PBS, pH 7.4) at −20°C. Sections were thoroughly washed with 1X PBS, incubated in hot 0.1 M sodium citrate buffer (pH 6.8) for antigen retrieval for 30 min, thoroughly rinsed with PBS, and then blocked in 5% bovine serum albumin (BSA) in TBST (1% Tween‐20 in 1X tris‐buffered saline, pH 7.4). Sections were then incubated with primary antibodies for targets of interest, anti‐GFAP (mouse, 1:1000; Cell Signaling, #3670), anti‐Iba1 (rabbit, 1:1000; FUJIFILM Wako, 019–19,741), and anti‐Iba1 (mouse, 1:500; Sigma‐Aldrich, MABN92) overnight in 1% BSA/TBST at 4°C followed by secondary incubation with appropriate AlexaFluor secondary antibodies (1:1000) at room temperature (RT). Nuclei were stained using DAPI (1:2000; Thermo Scientific). Immunolabeled sections were mounted with Prolong Gold with DAPI, and Z‐stack images were obtained using a Nikon W1CSU‐SoRa spinning disk confocal microscope. Images were denoised and 3D deconvolved using NIS‐Elements AR software (Nikon).

Images were analyzed to determine cell numbers and morphology using FIJI‐ImageJ 2. Cell numbers were obtained by quantifying the number of GFAP^+^ or Iba1^+^ cell bodies normalized to the total cell number based on DAPI labeling in each image. Cell numbers were determined from at least three images from 2 to 4 sections per animal. Astrocyte morphology was assessed via Sholl analysis using the Simple Neurite Tracer v4.2.1 plugin as described previously (Baier et al. 2022). Image Z‐stacks captured using a 40x objective were flattened to obtain maximum intensity projections. Individual GFAP‐labeled astrocytes from comparable regions of CA1 were traced for Sholl analysis (radius step size of 2 μm). GFAP‐labeled cells associated with blood vessels or overlapping with multiple nuclei (identified via DAPI stain) were excluded from analysis. Maximum branch lengths and maximal number of intersections were recorded for each astrocyte as described previously (Baier et al. 2022). A minimum of 30 CA1 GFAP‐labeled astrocytes derived from three to four sections were analyzed for each animal.

To visualize p16^INK4a^, tyramide signal amplification was used. Endogenous peroxidase activity of sections was quenched with 0.3% H_2_O_2_ in 1X PBS for 5 min, thoroughly washed, then blocked in 1X PBS containing 0.5% Triton X‐100 and 3% normal goat serum. Sections were incubated overnight with primary antibody against p16^INK4a^ (rabbit, 1:20,000; Abcam, ab211542), followed by washing in 1X PBS and secondary incubation with peroxidase‐conjugated AffiniPure goat anti‐rabbit antibody for 2 h at room temperature in a humidity chamber. Tyramide signal amplification (Faget and Hnasko 2015) was performed using a solution containing sulfo‐Cyanine5 tyramide (1:1000) and 0.003% H_2_O_2_ in 1X PBS for 10 min at RT. Residual HRP activity was then quenched using 0.3% H_2_O_2_. Sections were then blocked and fluorescently immunolabeled for cell type‐specific markers as described above. A minimum of two sections from *n* = 4–5 mice/group were analyzed.

### 4.5 Senescence‐Associated β‐Galactosidase (SA‐β‐Gal) Staining

Frozen brain sections (30 μm) were stained for SA‐β‐gal as described (Yousefzadeh et al. 2019). Briefly, sections were washed in 1X PBS and incubated in staining solution overnight (approx. 18 h) in a 37°C, CO_2_‐free incubator. The staining solution comprised potassium ferrocyanide (5 mM), potassium ferricyanide (5 mM), sodium chloride (150 mM), magnesium chloride (2 mM), and 5‐bromo‐4‐chloro‐indoly‐β‐D‐galactoside (X‐gal; 1 mg/mL) in citric acid/sodium phosphate buffer at pH 6.0. Following SA‐β‐gal staining, sections were rinsed in PBS and fluorescently immunolabeled for cell type‐specific markers. Dual bright‐field and confocal Z‐stack images were simultaneously captured using a Nikon Eclipse Ti2 microscope. Analysis was performed using FIJI following color deconvolution and image thresholding, with data presented as the average percent of SA‐β‐gal^+^ area from at least three sections per animal.

### 4.6 Administration of Senolytic Drugs

Dasatinib and quercetin were administered in aged C57Bl/6 mice for 8 weeks beginning at 22 months of age. A combination of dasatinib (5 mg/kg/day) and quercetin (50 mg/kg/day) dissolved in vehicle (60% Phosal 50 PG, 10% ethanol, 40% polyethylene glycol‐400) was administered via oral gavage for three consecutive days every 2 weeks over 2 months (Chaib et al. 2022; Thadathil et al. 2022). Control aged animals were administered vehicle alone. Gavages were performed consistently between 1:00 and 3:00 pm. The final dose of senolytic was given 1 week prior to cognitive assessment in the PhenoTyper at 24 months of age.

### 4.7 Cytokine and Chemokine PCR Array

cDNA was synthesized from 1000 ng of total hippocampal RNA using the RT^2^ First Strand kit (Qiagen) and utilized for the Real‐time RT^2^ Profiler PCR Mouse Cytokines and Chemokines array (PAMM‐150Z, Qiagen) according to the manufacturer's instructions. This array targets 84 cytokines, chemokines, interleukins, interferons, and growth factor genes associated with inflammation. Quantitative PCR was performed using RT^2^ SYBR Green ROX qPCR master mix (Qiagen) on the QuantStudio 12K Flex System (Applied Biosystems). Data were normalized to the geometric mean of five housekeeping genes (*Actb*, *B2m*, *Gapdh*, *Gusb*, *Hsp90ab1*) and analyzed as the following comparisons: young vs. intact, intact vs. impaired, impaired vs. senolytic. We utilized *a* = 0.05 as the threshold for significance between groups. Expression from a total of *n* = 4 young, intact, impaired, and senolytic hippocampi were analyzed per group.

### 4.8 High‐Resolution Respirometry

Hippocampal oxygen consumption was assessed using the Oroboros O2k‐FluoRespirometer as described previously (Logan et al. 2023) and according to our published protocol (Logan et al. 2024). Following permeabilization with saponin and washes, dorsal hippocampi (2.0 mg) were placed in the respirometer at 37°C with assay buffer containing 1 U/mL horseradish peroxidase and 5 U/mL SOD. Rates of respiration were determined following the sequential addition of the following substrates and inhibitors: glutamate (10 mM), malate (2 mM), pyruvate (5 mM), ADP (1 μM, 50 μM, and 2.5 mM), succinate (10 mM), rotenone (0.5 μM), TMPD (0.5 mM), and ascorbate (2 mM).

### 4.9 Statistical Analyses

All experiments were performed in multiple independent replicates per group as described. Statistical analyses were performed using GraphPad Prism 10, JMP v15.2.0 (SAS Inc.), and RStudio (build 375, R version 4.1). Behavioral data were analyzed using an age × group × time repeated measures ANOVA using JMP as previously reported (Logan et al. 2023). Molecular data were analyzed using a one‐way or two‐way ANOVA with Šidák's multiple comparisons test (parametric) or Kruskal‐Wallis test with Dunn's multiple comparisons (nonparametric) between comparisons of interest, as appropriate. Pearson correlation analysis was performed to assess relationships between behavioral and molecular data, with significance adjusted using the Benjamini‐Hochberg correction. Data are represented as mean ± SEM. Sample sizes are noted for each experiment with significance denoted as *p < 0.05, **p < 0.01, ***p < 0.001, ****p < 0.0001.

## Author Contributions

M.P.B. and S.L. designed the study. M.P.B., R.R., J.L.W., M.A.S., A.M.M., Z.T., and D.M.S. performed research. M.P.B., D.B.O., and S.L. analyzed data. M.P.B. and S.L. wrote the paper. M.P.B., D.M.S., and S.L. edited the paper. All authors approved the final manuscript.

## Conflicts of Interest

The authors declare no conflicts of interest.

## Supporting information


Appendix S1.

## Data Availability

The data that support the findings of this study are available in the manuscript and Appendix S1 of this study. Correspondence and requests for information should be addressed to S.L.
