# Supplementary material for: Cellular Senescence Is a Central Driver of Cognitive Disparities in Aging
Source: Aging Cell. 2025 Mar 12;24(6):e70041. doi: 10.1111/acel.70041 (PMC12151884; doi:10.1111/acel.70041)
Supplement: Supplementary file 1 — Appendix S1. [file ACEL-24-e70041-s001.pdf]

Supporting Information

Baier et. al, "Cellular senescence is a central driver of cognitive disparities in aging.", *Aging Cell*.

METHODS

Radial arm water maze

Hippocampal-dependent spatial learning was assessed in C57Bl/6N mice using a working memory task in a radial arm water maze (RAWM), as described in our previous studies in mice (Logan et al., 2018) and others in aged rats (Shukitt-Hale et al., 2004). The RAWM consisted of an 8-arm maze (67 cm in diameter) filled to two-thirds capacity with water made opaque using white food coloring. Mice were randomly placed in one arm of the maze and given four 60-second trials per day (starting at 0900hr) to locate a submerged hidden platform in one of the arms. If a mouse failed to find the platform within 60 seconds, it was guided to the platform by the experimenter.

Animal movements were recorded and analyzed using an automated tracking system (Noldus Ethovision XT 14, Wageningen, Netherlands). Key metrics recorded included the number of errors (entries into incorrect arms), path length (cm; total distance traveled to the target), velocity, and latency (time to reach the target). An error was recorded if the mouse traversed at least two-thirds of the length of an incorrect arm. Non-moving duration (NMD) was also recorded to confirm task engagement. Animals that did not move in the maze for >20 seconds (1/3rd of the total trial time) or more were not included in the analysis.

On day 1, mice underwent an initial learning phase to locate the submerged platform. On day 2, the platform was moved to a different arm (reversal), creating a short-term working memory paradigm (Light et al., 2010). RAWM data were subsequently analyzed using GraphPad Prism 10, with significance between young and aged groups determined via two-way ANOVA.

Primary astrocyte cultures

Primary astrocytes from mouse mixed cortical and hippocampal cultures were established from postnatal day 1-3 C57Bl/6J pups as previously described (Baier et al., 2022). Single-cell suspensions were generated via enzymatic digestion with papain and trituration. Cells were initially seeded on 50 µg/ml poly-D-lysine-coated plates in Neurobasal medium (Gibco) containing 2% NuSerum (Corning), penicillin (10 U/ml), and streptomycin (10 µg/ml). Astrocytes were split on DIV 7 and seeded at a density of $1.5x10^6$ cells / 10 cm^2. To induce reactive astrogliosis, confluent (~90%) astrocyte cultures were treated with the cytokine cocktail Il-1α (3 ng/ml), TNF (30 ng/ml), and C1q (400 ng/ml) (Liddelow et al., 2017) in serum-free media or vehicle for 24 hours. Prior to treatment, astrocytes were incubated in serum-free medium for 24 hours. Following treatment, cultures were rinsed with ice cold 1X PBS (pH 7.4), cell scraped, spun at 2000 x g, and cell pellets were flash frozen in liquid nitrogen and stored in -80°C until further analysis.

Body composition

Body composition measurements were performed using magnetic resonance (Echo MRI-100H Whole Body Composition Analyzer). Measurements were obtained for body fat and lean muscle and presented as the percent of body weight in grams.

Frailty index

We performed a frailty assessment prior to cognitive testing as previously described (Whitehead et al., 2013). Mice were assessed using multiple physical parameters assessing integument, musculoskeletal, vestibulocochlear, ocular, digestive, urogenital, and respiratory changes by three to four blinded assessors for each animal. Data are reported as the mean score of three to four assessors.

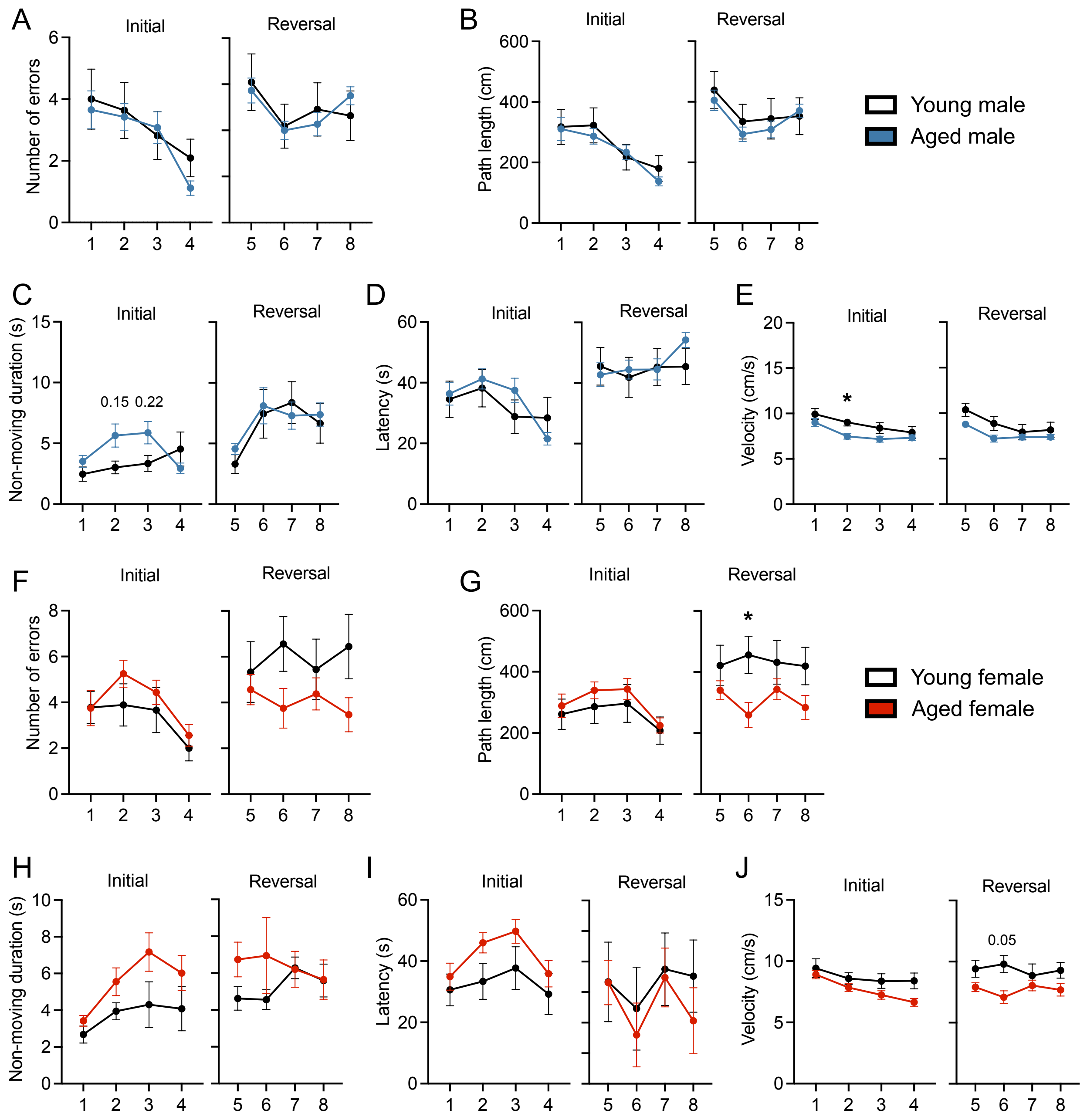

Figure S1. Radial arm water maze detected minimal age-related differences in male and female mice.

A-B. Young (5-7 mo) and aged (22-24mo) males made comparable number of errors (*A*) and path lengths to reach the target (*B*) during initial learning and reversal phases.
C-D. No statistically significant differences were detected in non-moving duration (*C*) or latency to reach the platform (*D*) between groups in males.
E. Velocity of aged male mice were statistically lower during trial 2 of initial learning, though no differences in velocity were noted at the end of initial or reversal learning.
F-J. No significant differences were noted between young and aged female mice for number of errors (*F*), non-moving duration (*H*), latency to platform (*I*), and velocity (*J*).

Aged females show reduced path length (*G*) during trial 2 of the reversal, but no overall differences were noted at the end of reversal learning phase.

For graphs *A*-*E*, colors represent the following: young male (black, *n*=11), aged male (blue, *n*=26). For graphs *F*-*J*, colors represent the following: young female (black, *n*=9), aged female (red, *n*=16). Error bars: mean ± SEM. $^*p<0.05$.

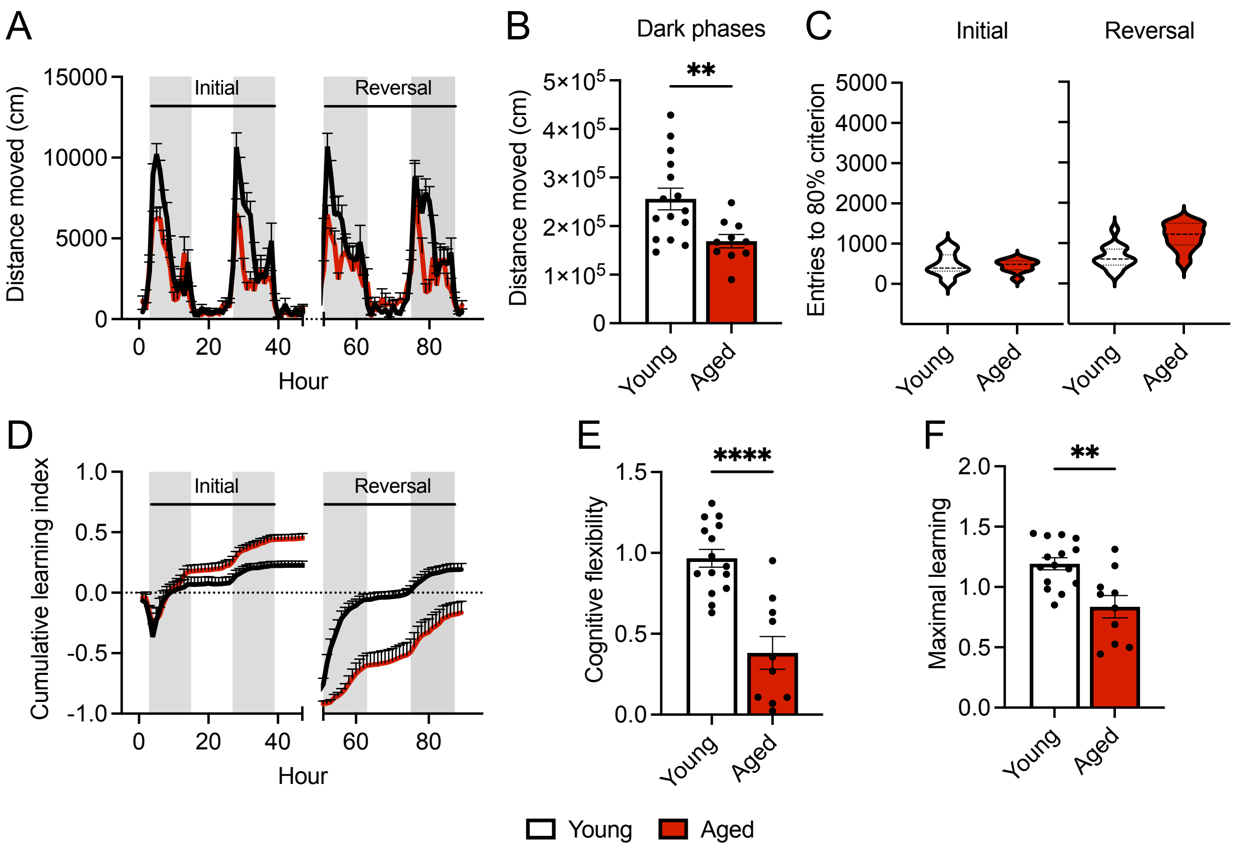

Figure S2. Age-related spatial memory deficits in 22-24mo female mice shows little heterogeneity in performance.

A. Circadian activity was comparable among young (5-7mo) and aged (22-24mo) female groups.
B. Total distance moved during the dark phases of the day/light cycle was significantly reduced in aged female mice.
C. Entries needed to reach 80% criterion in the initial learning and reversal learning tasks of young and aged female mice, with notable lack of heterogeneity in performance in the reversal phase.
D. Cumulative learning index depicts separations between young and aged females during the reversal phase.
E. Cognitive flexibility was assessed during the first ten hours of the reversal phase and was significantly reduced in the aged female group compared to young performance.
F. Maximal learning of aged cognitively impaired mice at the end of the reversal phase was significantly decreased compared to young.

For all graphs, colors represent the following: young (black, *n*=15), aged (red, *n*=10). Shaded bars in graphs *A* and *D* represent the dark periods of the L:D cycle. Error bars: mean ± SEM. $^{**}p<0.01$, $^{****}p<0.0001$.

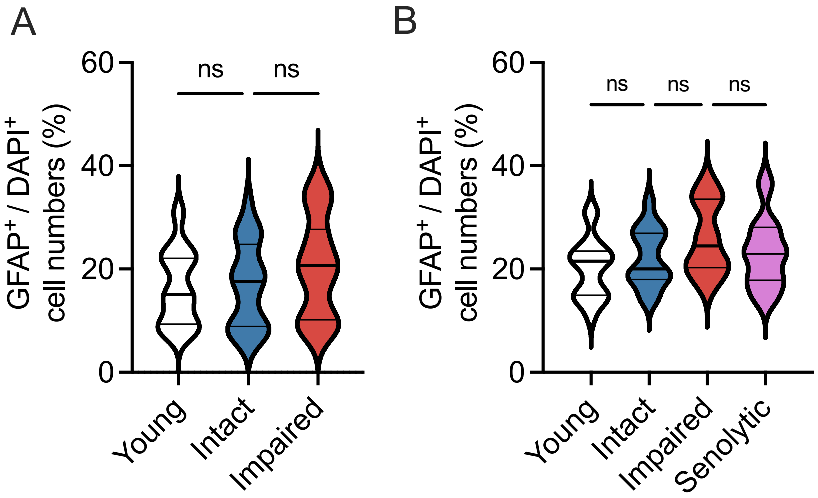

Figure S3. Astrocyte cell numbers were unchanged between cognitively stratified male mice.
A-B. The proportion of GFAP+ cell numbers normalized to total cell number (determined via $DAPI^+$ nuclei count) in the CA1 region of the hippocampus were unchanged in either cohorts of cognitively stratified aged male (*A*) or vehicle- and senolytic-treated males (*B*).

For all graphs, colors represent the following: young (black), cognitively intact (blue), cognitively impaired (red), senolytic (pink). A minimum of 30 CA1 $GFAP^+$-astrocytes derived from 3-4 sections were analyzed from *n=5* animals/group.

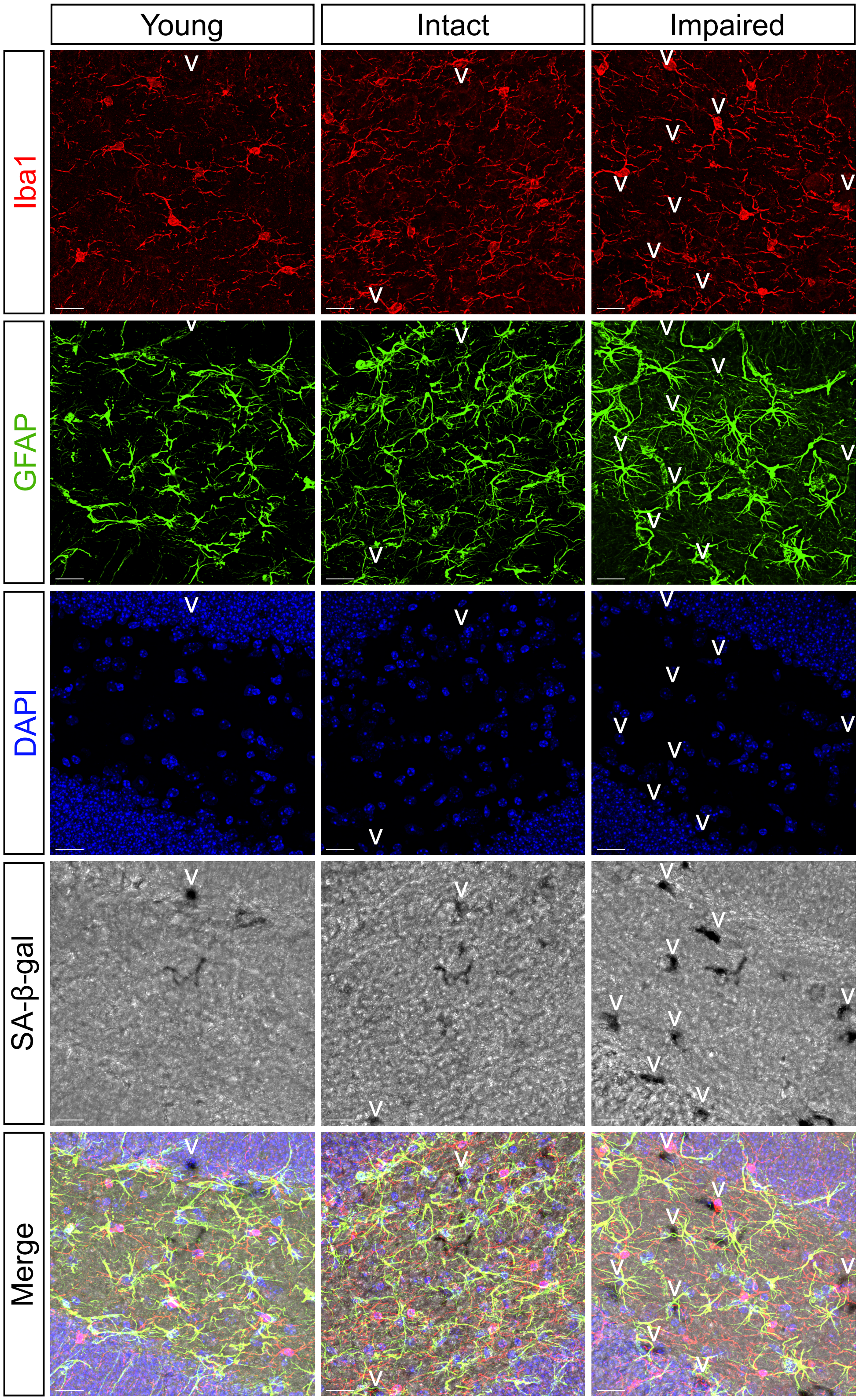

Figure S4. Representative images of SA-β-gal co-localization with microglial marker Iba1 and astrocyte marker GFAP within the dentate gyrus in cognitively stratified aged male mice. Scale bar, 20μm.

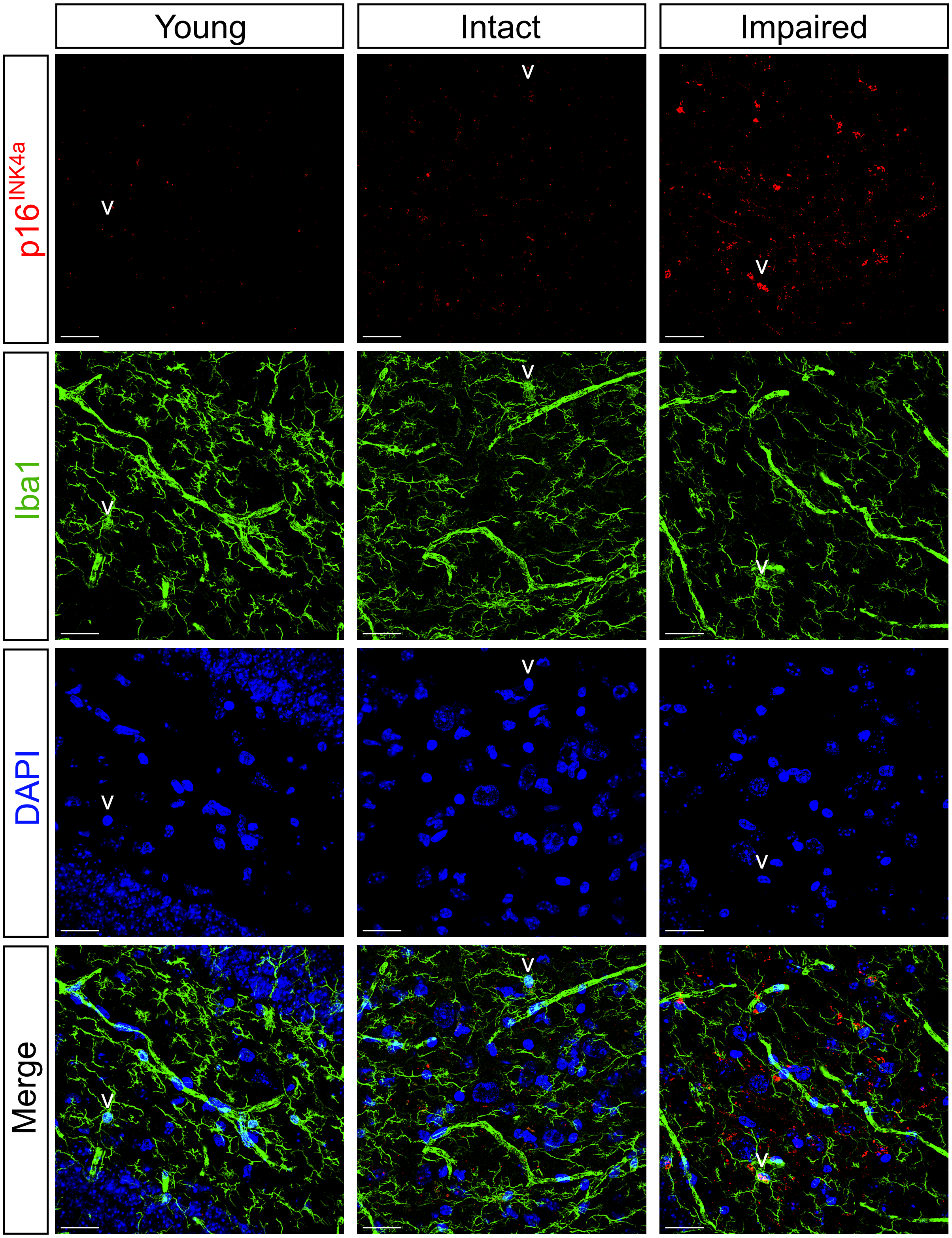

Figure S5. Representative images of p16^{INK4a} co-localization with microglial marker Iba1 within the dentate gyrus in cognitively stratified aged male mice. Arrows denoted cells depicted in Figure 3J. Scale bar, 20μm.

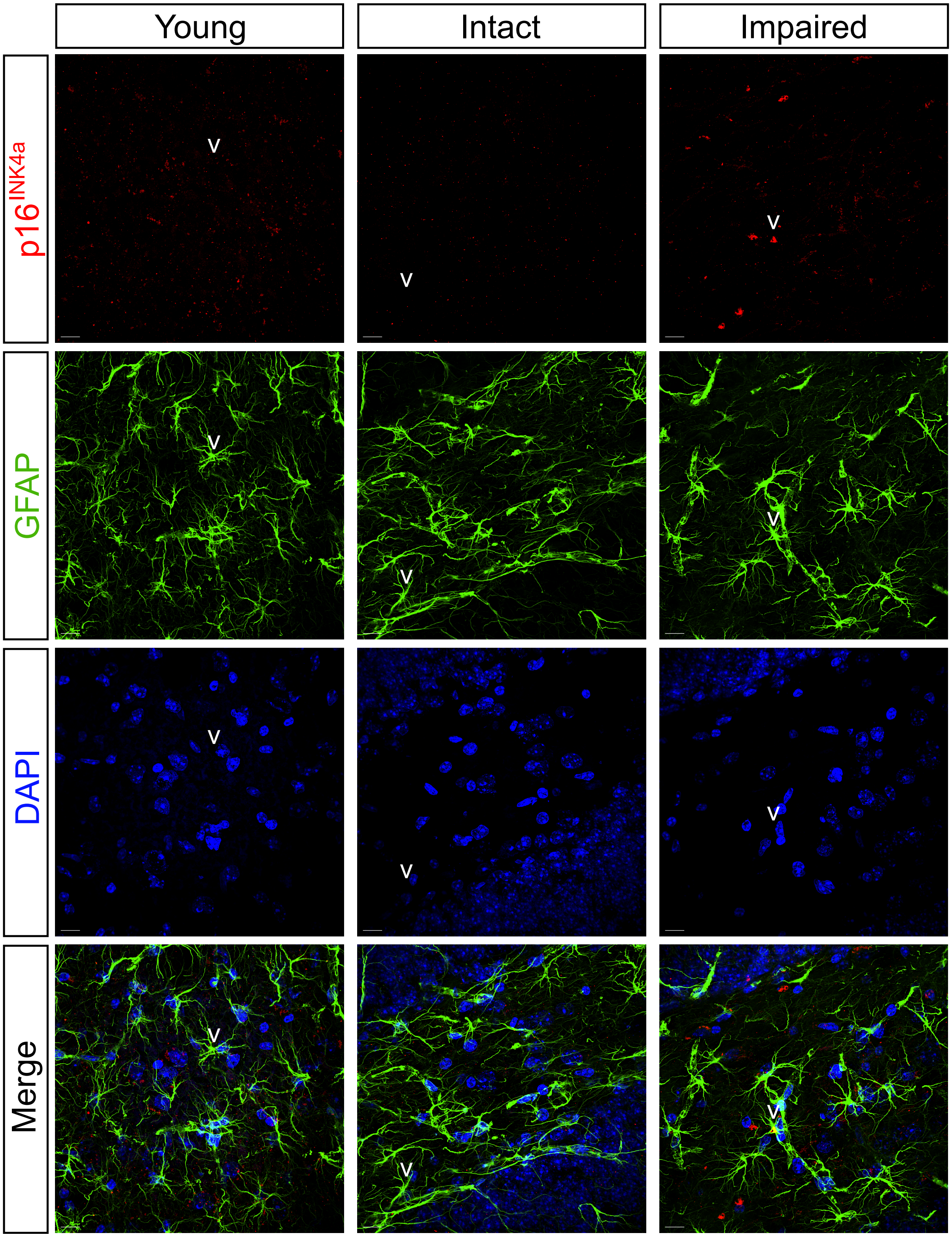

Figure S6. Representative images of p16^{INK4a} co-localization with astrocyte marker GFAP within the dentate gyrus in cognitively stratified aged male mice. Arrows denoted cells depicted in Figure 3J. Scale bar, 10µm.

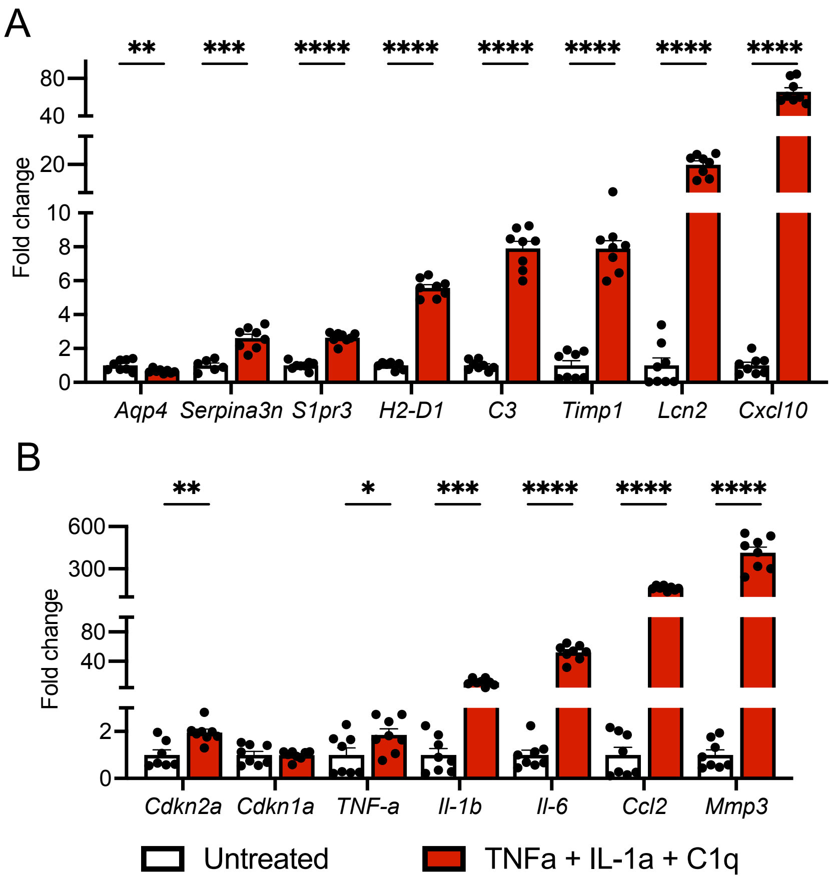

Figure S7. Sustained activation of reactive astrogliosis *in vitro* induces markers of cellular senescence.

A. Transcriptional expression of reactive astrogliosis markers in primary astrocytes cultured from day 1-3 post-natal C57 pups were confirmed following treatment with cytokine cocktail (TNF, IL-1 and C1q).

B. Transcriptional expression of senescence markers in primary astrocytes cultured from day 1-3 post-natal C57 pups were confirmed following treatment with IL-1α, TNF, and C1q.

For all graphs, colors represent the following: untreated (black), cytokine-treated (red). *n*=7-8 independent cultures/group. Error bars: mean ± SEM. *p<0.05, **p<0.01, ***p<0.001, ****p<0.0001.

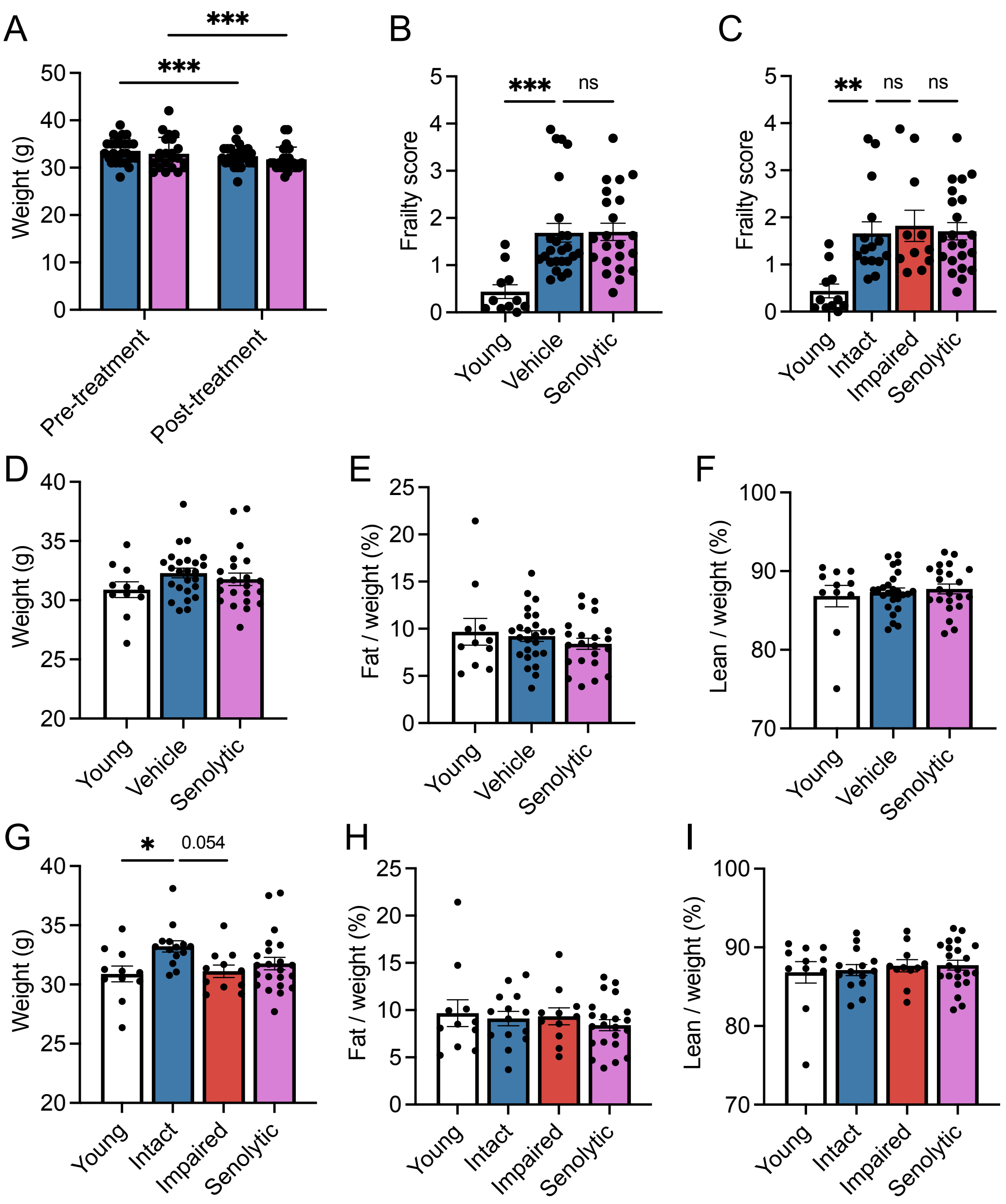

Figure S8. Senolytic treatment had no effect on frailty characteristics or body composition of aged male animals.

A. Body weights prior to and following completion of treatment were comparable between vehicle- and senolytic-treated male mice. A modest (4%) decrease in weight was noted for both groups at the conclusion of treatments in males.

B-C. Age-related frailty scores were unchanged with senolytic-treatment and did not differentiate cognitive status.

D-I. Body composition assessed via NMR was comparable among vehicle-treated, cognitively stratified and senolytic treated-aged mice with respect to percent fat and percent lean mass in both unstratified (*D-F*) and cognitively stratified vehicle groups (*G-I*).

For graphs *A*, *B*, *D-F*, colors represent the following: young (black), vehicle-treated (red), and senolytic-treated (pink). For graphs *C*, *G-I*, colors represent the following: young (black), vehicle-treated intact (blue), vehicle-treated impaired (red), senolytic-treated (pink). *n*=11 young, 25 vehicle-treated, and 22 senolytic-treated male mice. Error bars: mean ± SEM. *p<0.05, **p<0.01, ***p<0.001.

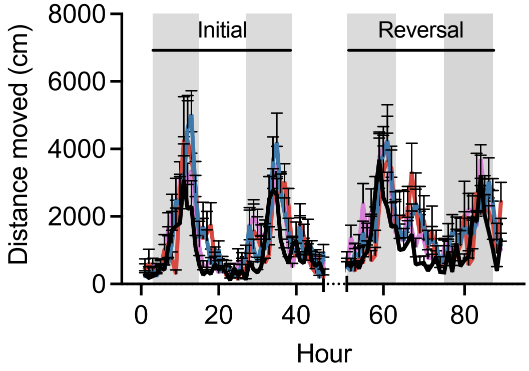

Figure S9. Circadian activity of senolytic-treated male mice was unchanged.

Circadian activity in the PhenoTyper assessed via onset of activity was comparable among young, vehicle-treated cognitively stratified, and senolytic-treated male mice. Shaded bars in graphs *A* and *D* represent the dark periods of the L:D cycle.

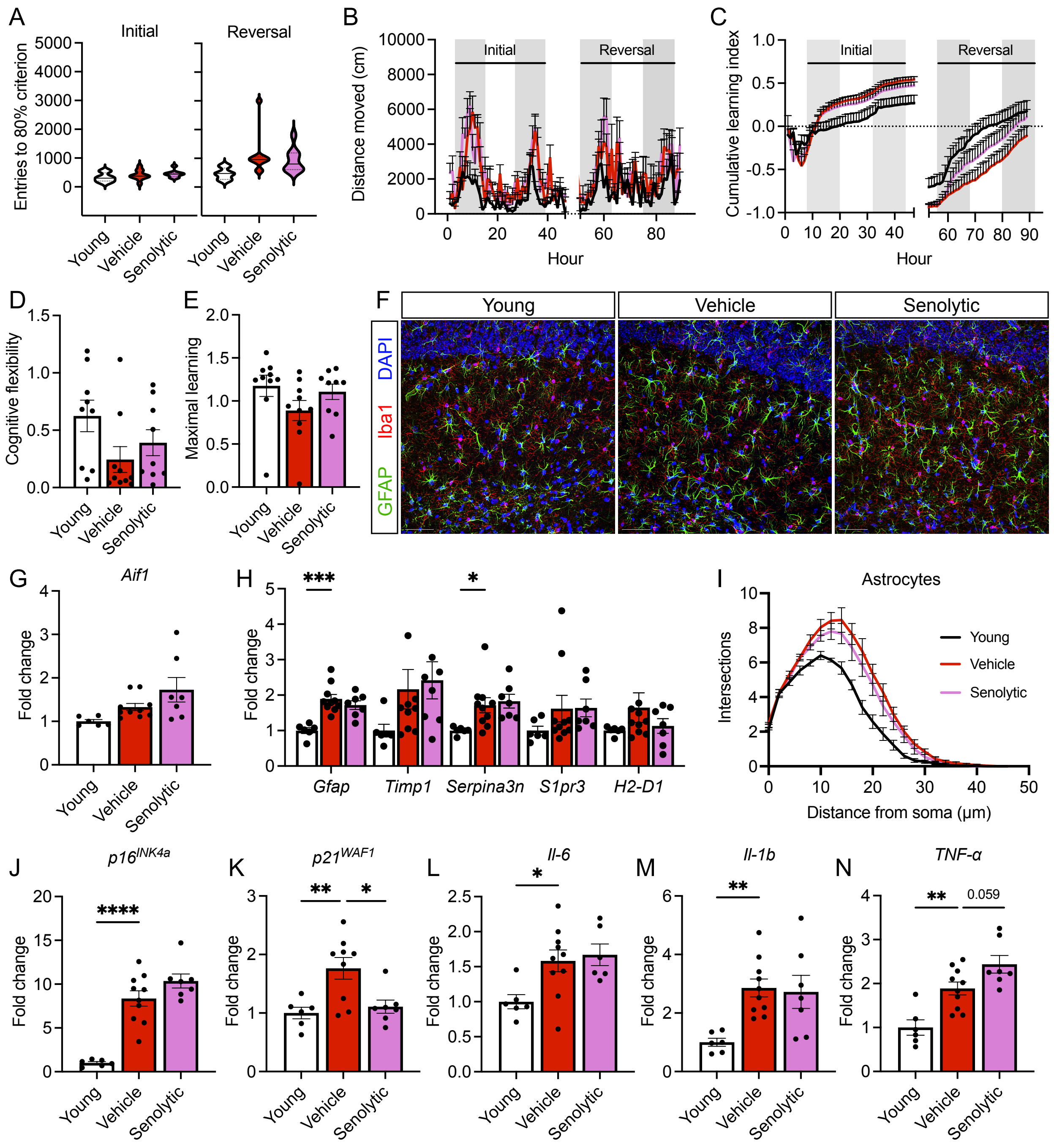

Figure S10. Senolytic treatment had little effect on spatial working memory impairments and gliosis and senescence markers in aged female mice.

A. Entries needed to reach 80% criterion in the initial learning and reversal learning tasks of young, vehicle-, and senolytic-treated female mice.

B. Circadian activity assessed via onset of activity was comparable among young (*n*=9), vehicle-treated (*n*=10), and senolytic-treated (*n*=9) female mice.

C. Cumulative learning index depicts similar performance between vehicle-treated and senolytic-treated female mice during both initial and reversal phases of the PhenoTyper.

D-E. Cognitive flexibility and maximal learning during the reversal was modestly increased in senolytic-treated aged female mice compared to vehicle-treated controls
F. Representative confocal images of immunostained hippocampal sections within the CA1 region labeled for GFAP (green; astrocytes), Iba1 (red; microglia), and DAPI (blue; nuclei) from cognitively stratified mice. Scale bar, 50µm.
G. Transcriptional expression of microglial marker *Aif1* in the hippocampus. n=6-9 animals/group.
H. Transcriptional expression of astrocyte cytoskeletal element *Gfap* and astrocyte reactivity markers (*Timp1, Serpina3n, S1pr3,* and *H2-D1*) within the hippocampus. n=5 mice/group.
I. Quantification of the number of $GFAP^+$ astrocyte projections plotted against the radial distance from the soma obtained via Sholl analysis in the CA1 region of the hippocampus.
J-N. Transcriptional expression of cell cycle arrest markers ($p16^{INK4a}$ and $p21^{WAF1}$) and senescence-associated secretory phenotype factors (*Il-6*, *Il-1b*, *Tnf-α*) within the hippocampus*.* n=6-9 animals/group.

For all graphs, colors represent the following: young (black, *n*=9), vehicle-treated (red, *n*=10), and senolytic-treated (pink, *n*=9). Shaded bars in graphs B-C represent the dark periods of the L:D cycle. Error bars: mean ± SEM. *p<0.05, **p<0.01, ***p<0.001, ****p<0.0001.

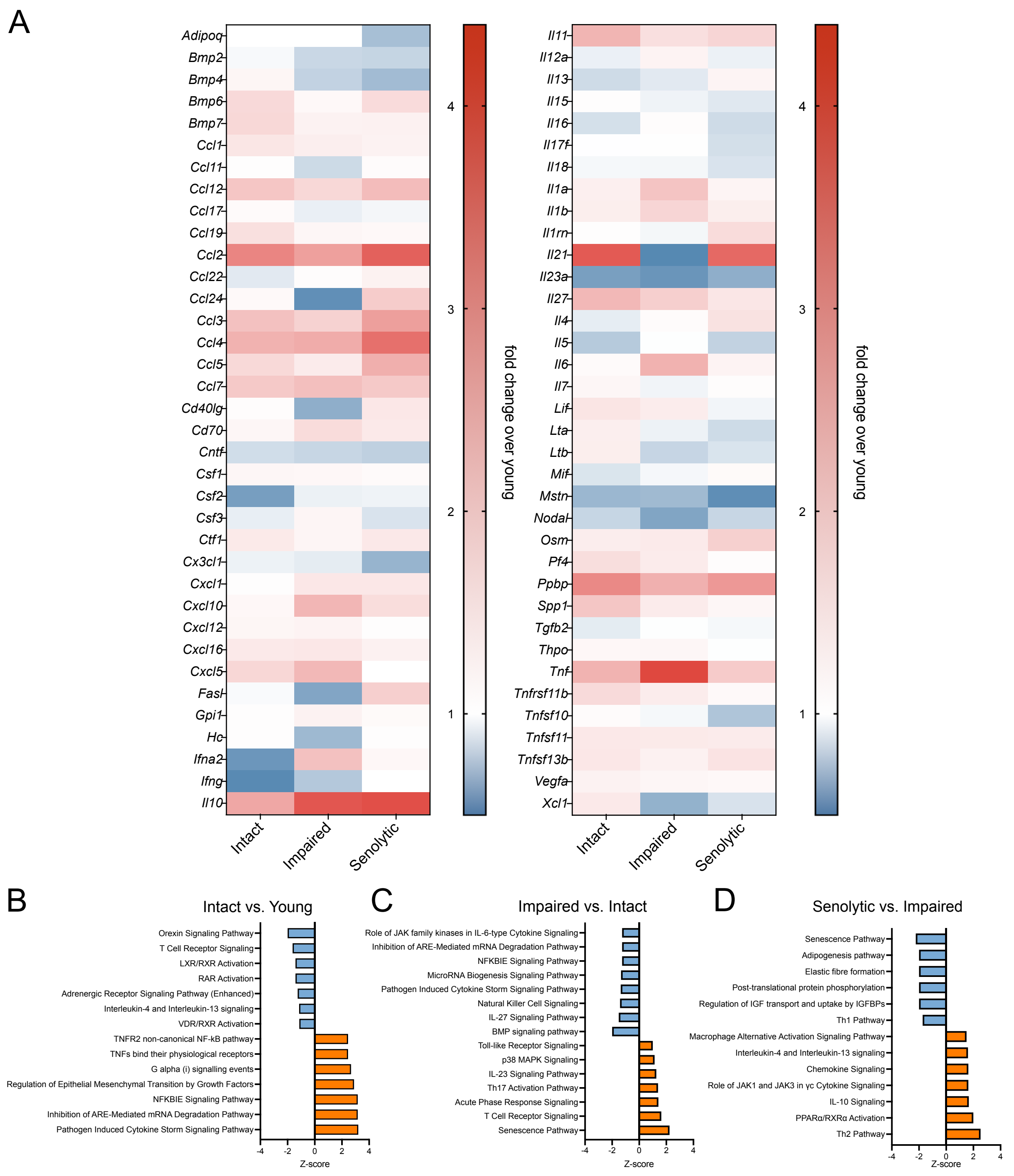

Figure S11. Transcriptional profile of cytokines and chemokines in the hippocampus.

A. Expression heat map for the transcript level of cytokines and chemokines in the hippocampus of vehicle- and senolytic-treated mice. Data are represented as the group fold change over young. n=4 mice per group.

B-D. Top upregulated and downregulated pathways between comparisons as identified by Ingenuity Pathway Analysis ($p<0.05$).

Male		Pearson *r*	R^2	p-value	p-adjusted
p16INK4a	***Il6***	**0.7183**	**0.5160**	**0.0012**	**0.0035**
p16INK4a	***Gfap***	**0.8829**	**0.7796**	**2.68E-06**	**5.62E-05**
p16INK4a	***Tnf***	**0.8597**	**0.7390**	**9.73E-06**	**9.12E-05**
p16INK4a	***Aif1***	**0.8108**	**0.6574**	**0.0004**	**0.0020**
p16INK4a	Initial ETC80%	-0.2492	0.0621	0.3903	0.4258
p16INK4a	**Reversal ETC80%**	**0.8050**	**0.6481**	**9.69E-05**	**0.0005**
p16INK4a	**Cognitive flexibility**	**-0.7038**	**0.4954**	**0.0016**	**0.0042**
p16INK4a	**Distance moved**	**0.6358**	**0.4043**	**0.0061**	**0.0122**
Il6	***Gfap***	**0.6388**	**0.4081**	**0.0058**	**0.0122**
Il6	*Tnf*	0.4249	0.1805	0.0891	0.1146
Il6	***Aif1***	**0.9203**	**0.8469**	**3.12E-06**	**5.62E-05**
Il6	Initial ETC80%	-0.3739	0.1398	0.1879	0.2182
Il6	Reversal ETC80%	0.5021	0.2521	0.0400	0.0576
Il6	**Cognitive flexibility**	**-0.6268**	**0.3929**	**0.0071**	**0.0131**
Il6	**Distance moved**	**0.7031**	**0.4944**	**0.0016**	**0.0042**
Gfap	***Tnf***	**0.7448**	**0.5547**	**0.0006**	**0.0024**
Gfap	***Aif1***	**0.8976**	**0.8058**	**1.33E-05**	**9.12E-05**
Gfap	Initial ETC80%	-0.3930	0.1544	0.1645	0.1974
Gfap	**Reversal ETC80%**	**0.8505**	**0.7234**	**1.52E-05**	**9.12E-05**
Gfap	**Cognitive flexibility**	**-0.6151**	**0.3783**	**0.0086**	**0.0141**
Gfap	**Distance moved**	**0.6252**	**0.3909**	**0.0073**	**0.0131**
Tnf	*Aif1*	0.5543	0.3073	0.0397	0.0576
Tnf	Initial ETC80%	-0.1357	0.0184	0.6437	0.6617
Tnf	**Reversal ETC80%**	**0.6195**	**0.3838**	**0.0080**	**0.0137**
Tnf	Cognitive flexibility	-0.4865	0.2366	0.0477	0.0636
Tnf	Distance moved	0.4200	0.1764	0.0932	0.1157
Aif1	Initial ETC80%	-0.2668	0.0712	0.4277	0.4528
Aif1	**Reversal ETC80%**	**0.8981**	**0.8065**	**1.30E-05**	**9.12E-05**
Aif1	**Cognitive flexibility**	**-0.7923**	**0.6277**	**0.0007**	**0.0026**
Aif1	**Distance moved**	**0.6918**	**0.4786**	**0.0061**	**0.0122**
Initial ETC80%	Reversal ETC80%	-0.3104	0.0963	0.2802	0.3152
Initial ETC80%	Cognitive flexibility	0.5483	0.3006	0.0423	0.0586
Initial ETC80%	Distance moved	-0.1284	0.0165	0.6617	0.6617
**Reversal ETC80%**	**Cognitive flexibility**	**-0.7305**	**0.5337**	**0.0009**	**0.0028**
**Reversal ETC80%**	**Distance moved**	**0.6489**	**0.4211**	**0.0048**	**0.0116**
Cognitive flexibility	Distance moved	-0.5184	0.2688	0.0330	0.0517

Table S1. Measures of cognitive status correlate with transcriptional expression of cellular senescence, neuroinflammation, and gliosis markers in male mice. Simple linear regression of behavioral metrics (entries to 80% criterion [ETC80%] for

initial and reversal phases, cognitive flexibility, distance moved) and transcriptional expression of cellular senescence, neuroinflammation, and gliosis markers for male mice. Pearson correlation coefficient *r*, R^2, p-values, and adjusted p-values are reported for each comparison. Significant regressions of p-adjusted < 0.05 are bolded.

Female		Pearson *r*	R^2	p-value	p-adjusted
p16^{INK4a}	***Il1b***	**0.6314**	**0.3987**	**0.0009**	**0.0070**
p16^{INK4a}	*Il6*	0.3347	0.1120	0.1099	0.2557
p16^{INK4a}	***Tnf***	**0.8876**	**0.7878**	**7.38E-09**	**1.66E-07**
p16^{INK4a}	***Gfap***	**0.8339**	**0.6954**	**4.16E-07**	**6.23E-06**
p16^{INK4a}	*Aif1*	0.4007	0.1606	0.0523	0.1807
p16^{INK4a}	Initial ETC80%	0.0807	0.0065	0.7079	0.9369
p16^{INK4a}	Reversal ETC80%	-0.0530	0.0028	0.8058	0.9574
p16^{INK4a}	Cognitive flexibility	-0.4306	0.1854	0.0357	0.1606
p16^{INK4a}	Maximal learning	-0.3948	0.1559	0.0562	0.1807
Il1b	*Il6*	-0.0413	0.0017	0.8480	0.9574
Il1b	***Tnf***	**0.5977**	**0.3572**	**0.0020**	**0.0131**
Il1b	***Gfap***	**0.6595**	**0.4349**	**0.0005**	**0.0049**
Il1b	*Aif1*	0.0365	0.0013	0.8654	0.9574
Il1b	Initial ETC80%	0.0133	0.0002	0.9506	0.9574
Il1b	Reversal ETC80%	-0.0206	0.0004	0.9237	0.9574
Il1b	Cognitive flexibility	-0.0618	0.0038	0.7741	0.9574
Il1b	Maximal learning	-0.1267	0.0160	0.5554	0.8062
Il6	*Tnf*	0.3314	0.1098	0.1137	0.2557
Il6	*Gfap*	0.1024	0.0105	0.6340	0.8915
Il6	***Aif1***	**0.9283**	**0.8618**	**6.31E-11**	**2.84E-09**
Il6	Initial ETC80%	0.2109	0.0445	0.3225	0.5582
Il6	Reversal ETC80%	-0.0162	0.0003	0.9401	0.9574
Il6	Cognitive flexibility	-0.3679	0.1353	0.0770	0.2176
Il6	Maximal learning	-0.3598	0.1295	0.0842	0.2228
Tnf	***Gfap***	**0.6525**	**0.4258**	**0.0005**	**0.0049**
Tnf	*Aif1*	0.3674	0.1350	0.0774	0.2176
Tnf	Initial ETC80%	0.1455	0.0212	0.4976	0.7464
Tnf	Reversal ETC80%	-0.1623	0.0263	0.4486	0.6962
Tnf	Cognitive flexibility	-0.2562	0.0656	0.2269	0.4255
Tnf	Maximal learning	-0.2030	0.0412	0.3415	0.5692
Gfap	*Aif1*	0.0901	0.0081	0.6754	0.9210
Gfap	Initial ETC80%	-0.0115	0.0001	0.9574	0.9574
Gfap	Reversal ETC80%	0.0354	0.0013	0.8696	0.9574
Gfap	Cognitive flexibility	-0.4876	0.2378	0.0156	0.0782
Gfap	Maximal learning	-0.3962	0.1570	0.0553	0.1807
Aif1	Initial ETC80%	0.2370	0.0562	0.2648	0.4767
Aif1	Reversal ETC80%	-0.0296	0.0009	0.8907	0.9574
Aif1	Cognitive flexibility	-0.2892	0.0837	0.1704	0.3486
Aif1	Maximal learning	-0.2986	0.0892	0.1563	0.3350
Initial ETC80%	Reversal.ETC80	-0.2639	0.0697	0.2127	0.4162
Initial ETC80%	Cognitive flexibility	0.0442	0.0020	0.8375	0.9574
Initial ETC80%	Maximal learning	-0.1629	0.0265	0.4469	0.6962
Reversal ETC80%	Cognitive flexibility	-0.3963	0.1570	0.0552	0.1807
Reversal ETC80%	Maximal learning	-0.3449	0.1190	0.0988	0.2471
**Cognitive flexibility**	**Maximal learning**	**0.5801**	**0.3365**	**0.0030**	**0.0167**

Table S2. Correlation of behavioral metrics with transcriptional expression of cellular senescence, neuroinflammation, and gliosis markers in female mice. Simple linear regression of behavioral metrics (entries to 80% criterion [ETC80%] for initial and reversal phases, cognitive flexibility, maximal learning during the reversal phase) and transcriptional expression of cellular senescence, neuroinflammation, and gliosis markers for female. Pearson correlation coefficient *r*, R^2, p-values, and adjusted p-values are reported for each comparison. Significant regressions of p-adjusted < 0.05 are bolded.

REFERENCES

Baier, M. P., Nagaraja, R. Y., Yarbrough, H. P., Owen, D. B., Masingale, A. M., Ranjit, R., Stiles, M. A., Murphy, A., Agbaga, M. P., Ahmad, M., Sherry, D. M., Kinter, M. T., Van Remmen, H., & Logan, S. (2022). Selective Ablation of Sod2 in Astrocytes Induces Sex-Specific Effects on Cognitive Function, d-Serine Availability, and Astrogliosis. *J Neurosci*, *42*(31), 5992-6006. https://doi.org/10.1523/jneurosci.2543-21.2022

Liddelow, S. A., Guttenplan, K. A., Clarke, L. E., Bennett, F. C., Bohlen, C. J., Schirmer, L., Bennett, M. L., Münch, A. E., Chung, W. S., Peterson, T. C., Wilton, D. K., Frouin, A., Napier, B. A., Panicker, N., Kumar, M., Buckwalter, M. S., Rowitch, D. H., Dawson, V. L., Dawson, T. M.,…Barres, B. A. (2017). Neurotoxic reactive astrocytes are induced by activated microglia. *Nature*, *541*(7638), 481-487. https://doi.org/10.1038/nature21029

Light, K. R., Kolata, S., Wass, C., Denman-Brice, A., Zagalsky, R., & Matzel, L. D. (2010). Working memory training promotes general cognitive abilities in genetically heterogeneous mice. *Curr Biol*, *20*(8), 777-782. https://doi.org/10.1016/j.cub.2010.02.034

Logan, S., Pharaoh, G. A., Marlin, M. C., Masser, D. R., Matsuzaki, S., Wronowski, B., Yeganeh, A., Parks, E. E., Premkumar, P., Farley, J. A., Owen, D. B., Humphries, K. M., Kinter, M., Freeman, W. M., Szweda, L. I., Van Remmen, H., & Sonntag, W. E. (2018). Insulin-like growth factor receptor signaling regulates working memory, mitochondrial metabolism, and amyloid-β uptake in astrocytes. *Mol Metab*, *9*, 141-155. https://doi.org/10.1016/j.molmet.2018.01.013

Shukitt-Hale, B., McEwen, J. J., Szprengiel, A., & Joseph, J. A. (2004). Effect of age on the radial arm water maze-a test of spatial learning and memory. *Neurobiol Aging*, *25*(2), 223-229. https://doi.org/10.1016/s0197-4580(03)00041-1

Whitehead, J. C., Hildebrand, B. A., Sun, M., Rockwood, M. R., Rose, R. A., Rockwood, K., & Howlett, S. E. (2013). A Clinical Frailty Index in Aging Mice: Comparisons With Frailty Index Data in Humans. *The Journals of Gerontology: Series A*, *69*(6), 621-632. https://doi.org/10.1093/gerona/glt136
